# Molecular Dynamics Simulations of Phosphorylated Intrinsically Disordered Proteins: A Force Field Comparison

**DOI:** 10.3390/ijms221810174

**Published:** 2021-09-21

**Authors:** Ellen Rieloff, Marie Skepö

**Affiliations:** 1 Division of Theoretical Chemistry, Lund University, P.O. Box 124, SE-221 00 Lund, Sweden; ellen.rieloff@teokem.lu.se; 2 LINXS—Lund Institute of Advanced Neutron and X-ray Science, Scheelevägen 19, SE-223 70 Lund, Sweden

**Keywords:** intrinsically disordered proteins, phosphorylation, force fields

## Abstract

Phosphorylation is a common post-translational modification among intrinsically disordered proteins and regions, which helps regulate function by changing the protein conformations, dynamics, and interactions with binding partners. To fully comprehend the effects of phosphorylation, computer simulations are a helpful tool, although they are dependent on the accuracy of the force field used. Here, we compared the conformational ensembles produced by Amber ff99SB-ILDN+TIP4P-D and CHARMM36m, for four phosphorylated disordered peptides ranging in length from 14–43 residues. CHARMM36m consistently produced more compact conformations with a higher content of bends, mainly due to more stable salt bridges. Based on comparisons with experimental size estimates for the shortest and longest peptide, CHARMM36m appeared to overestimate the compactness. The difference between the force fields was largest for the peptide showing the greatest separation between positively charged and phosphorylated residues, in line with the importance of charge distribution. For this peptide, the conformational ensemble did not change significantly upon increasing the ionic strength from 0 mM to 150 mM, despite a reduction of the salt-bridging probability in the CHARMM36m simulations, implying that salt concentration has negligible effects in this study.

## 1. Introduction

Intrinsically disordered proteins (IDPs) are characterized by a lack of a tertiary structure under physiological conditions [1,2], which means that they are better described by an ensemble of different conformations than a single structure. This is reflected in their free energy landscapes, which normally are rather flat without a deep energy minimum as for globular proteins [3]. The flattened energy landscape makes IDPs very sensitive to changes in the environment and post-translational modifications (PTMs) of the sequence. A common type of reversible PTM is phosphorylation, which introduces extra negative charges and the possibility of forming hydrogen bonds and salt bridges [4]. Phosphorylation is commonly employed by cells as a regulatory mechanism, as it can change both the conformational ensemble and the dynamics, as well as the interaction with a binding partner, and therefore affect function. The functional implications of phosphorylation can be drastic, such as for the disordered neuroprotein tau, for which hyperphosphorylation has been related to amyloid fibril formation in Alzheimer’s disease [5]. In proteins such as statherin and caseins, the phosphorylated residues are essential for their ability to bind to the tooth surface [6,7] or sequester calcium [8].

Experimental techniques such as small-angle X-ray scattering (SAXS) and fluorescence resonance energy transfer (FRET) have been used to provide information on global conformational changes upon phosphorylation of intrinsically disordered proteins or regions, while circular dichroism spectroscopy and nuclear magnetic resonance (NMR) have detected changes in secondary structure or other local arrangements such as salt bridges [9,10,11,12,13,14]. However, due to the vast conformational ensembles possessed by IDPs, computer simulations are often a useful complement to obtain more detailed information, though this requires accurate models and force fields. We have previously shown that a coarse-grained “one bead per residue model” has proven to accurately predict average radius of gyration (R_g_) and scattering curves for various IDPs, including statherin, although producing overly compact conformations of other more phosphorylated IDPs [15]. The two-site UNRES model has recently been extended with parameters for phosphorylated residues [16] and applied to study phosphorylation-induced folding of an IDP [17]. Although coarse-grained models are more computationally efficient and generally easier to interpret than atomistic models, they can lack in detail. In atomistic modelling, there is continuous development of force fields and water models towards more accurately describing IDPs, and some important adjustment have been the refinement of the backbone dihedral angles and balancing the water–protein and protein–protein interactions; see for example the following reviews and references within [18,19]. However, we recently showed that while the commonly used force fields CHARMM36m and Amber ff99SB-ILDN+TIP4P-D accurately captured the global dimensions of the 15-residue-long N-terminal fragment of Statherin in the nonphosphorylated state, it overestimated the compactness in the phosphorylated state [20]. More recently, overcompaction was also observed for two approximately 80-residue-long phosphorylated IDPs in several force fields, where it was suggested to depend on an overestimation of charge–charge interactions [21], in line with an overstabilization of salt bridges in standard force fields [22]. In this study, we made a further comparison of the two aforementioned force fields, by applying them to four phosphorylated peptides, namely two different fragments from tau, specifically residues 173-183 (Tau1) and 225-246 (Tau2), the first 25 amino acids in the milk protein β-casein (bCPP) and the saliva protein statherin (Stath). For all peptides, CHARMM36m was shown to sample more compact conformations than Amber ff99SB-ILDN+TIP4P-D, associated with a much higher probability for salt bridges. The effect was more pronounced in sequences with large separation between phosphorylated residues and positively charged residues, showing the importance of charge distribution. In bCPP, which showed the largest differences between the force fields, the addition of 150 mM NaCl did not change the average size estimates and shape significantly, despite a significant reduction of salt bridge occurrence in CHARMM36m. This implies that salt bridges are still of importance at 150 mM salt and that we can ignore the effects of salt concentration in this study.

## 2. Results and Discussion

Four phosphorylated peptides, shown in Table 1, were simulated at physiological pH using two different force fields: Amber ff99SB-ILDN [23] with the TIP4P-D [24] water model and parameters for the phosphorylated residues from Homeyer et al. [25] and Steinbrecher et al. [26] (A99) and CHARMM36m [27] with the CHARMM-modified TIP3P water model [28] (C36). The peptides were chosen based on availability of experimental data to compare with and size considering the computational expense.

### 2.1. Size and Shape

**Figure 1:**
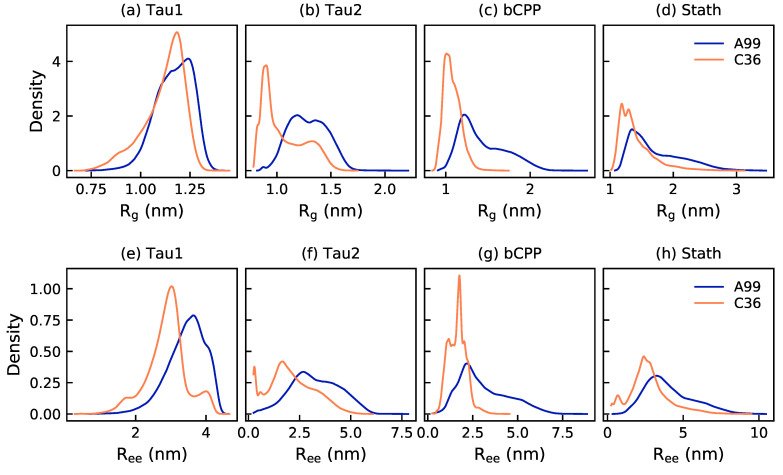
Distribution of the radius of gyration (**top row**) and the end-to-end distance (**bottom row**) of Tau1, Tau2, bCPP, and Stath simulated with Amber ff99SB-ILDN (A99) and CHARMM36m (C36). The legend applies to all panels.

For all four peptides, the two force fields produced different conformational ensembles, as seen by the distributions of the R_g_ and the end-to-end distance (R_ee_) in Figure 1. The C36 distributions were narrower and centered on values lower than the A99 distributions. For Tau2 and bCPP, the R_g_ distribution had a sharp peak at low values. From the average R_g_ and R_ee_ presented in Table 2, it is clear that Tau1 showed the smallest differences between the force fields, while bCPP showed the largest differences. The discrepancy was larger for R_ee_ than R_g_. For Tau1, Chin et al. [11] determined the average R_ee_ to be ∼3.17 nm, based on FRET. To obtain an R_ee_ distance distribution from the FRET data they assumed a semi-flexible polymer model, and the resulting distribution was skewed towards longer distances, with the peak value located at 3.64 nm (Figure 4A in ref. [11]). Comparing A99 and C36 to the experimental average, A99 overestimated it approximately as much as C36 underestimated it. However, the skewed shape and peak position at 3.64 nm produced in A99 was in better experimental agreement than C36, since the distribution in C36 was more symmetrical with multiple peaks and had the main peak located at 3.03 nm.

For Stath, earlier published SAXS data [15] provided an R_g_ of 1.93±0.2 nm; hence, R_g_ was 10% smaller in A99 and 27% smaller in C36. Since R_g_ determined from SAXS includes a hydration shell, it was expected that R_g_ calculated from simulations would be slightly smaller, although not to that extent. Since it is not straightforward which contrast to use for the hydration shell in the calculations of scattering curves for IDPs [29], in Supplementary Figure S1 and Table S2, we compared the curves calculated using different contrasts of the hydration shell to the experimental curve for Stath. While the highest contrast used (0.03 *e*/Å^3^) yielded the best agreement with the scattering curve, it provided the worst agreement with the Kratky plot. Henriques et al. [29] showed that the optimal contrast for IDPs was often between 0.01 *e*/Å^3^ and 0.02 *e*/Å^3^, although varying with both force field and protein. The optimal values for A99 and C36 were suggested to be around 0.0075 *e*/Å^3^ and 0.02 *e*/Å^3^, respectively. While the suggested optimal value gave reasonable agreement with the experimental form factor for A99, this was not the case for C36. For C36, all contrasts >0 clearly showed larger compaction than the experimental Kratky plot.

**Figure 2:**
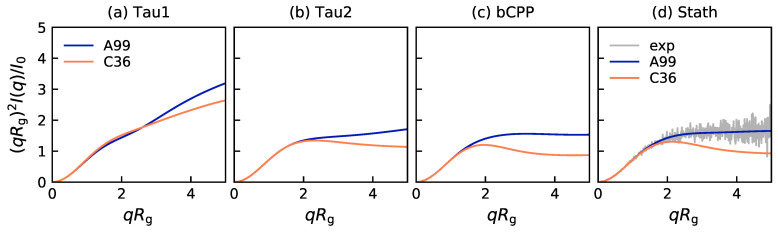
Dimensionless Kratky plot from simulations with Amber ff99SB-ILDN and CHARMM36m for (**a**) Tau1, (**b**) Tau2, (**c**) bCPP, and (**d**) Stath. In Panel (d), experimental data from Cragnell et al. [15] are included for comparison. The legend in Panel (a) is applicable to all panels.

Even without experimental scattering curves to compare to, the dimensionless Kratky plot, presented in Figure 2, is a good way of comparing the average shape of the peptides in the two different force fields. The short peptide Tau1 exhibited a more extended shape than the other three peptides, which in A99 were shown to have more of the typical IDP behavior, resembling a Gaussian chain. For all four peptides, the Kratky plot produced in C36 had a lower slope, and for the three longest peptides, the curve started to move towards the bell-shaped curve typical of globular proteins. Hence, this implies that C36 sampled more compact or well-defined conformations than A99, in accordance with the R_g_ and R_ee_ distributions. Notice also that the Kratky plot of Stath in A99 was in excellent agreement with the experimental data, while the curve corresponding to C36 fell below, as shown in Figure 2d.

### 2.2. Salt Bridges and Secondary Structure

**Figure 3:**
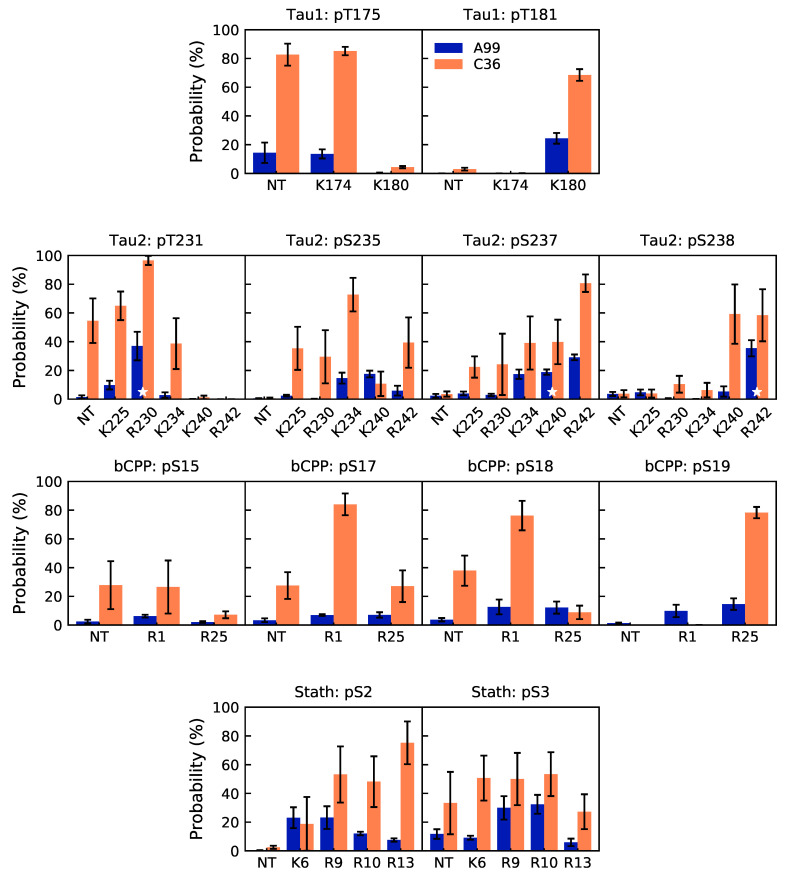
Probability of possible salt bridge interactions for the phosphorylated residues with the N-terminus (NT) and positively charged residues in Tau1 (**first row**), Tau2 (**second row**), bCPP (**third row**), and Stath (**last row**). For Tau2, experimentally established salt bridges [12] are marked with a white star. Error bars correspond to errors calculated by block averaging.

Since our previous study [20] suggested that overstabilized salt bridges are the reason why C36 produces more compact conformations than A99, we calculated the occupancy of the possible salt bridge interactions involving the phosphorylated residues. Figure 3 indeed shows that salt bridges were formed much more in C36 than A99, for all the peptides. In Tau2 and bCPP, the strong salt bridges in C36 restricted the conformational ensemble, which explains the smaller and narrower distributions of R_g_ and R_ee_. In bCPP, the salt-bridging residues were well separated in the sequence, therefore having a larger effect on the R_g_ and R_ee_ distributions. In Tau1, the salt bridge interactions almost exclusively appeared between the adjacent residues and between pT175 and the N-terminal.

For Tau2, there is experimental evidence of the following salt bridges, detected by NMR experiments: pT231–R230, pS237–K240, and pS238–R242 [12]. pT231–R230 and pS238–R242 are indeed two of the most often occurring salt bridges in A99, while pS237–R242 is more common than pS237–K240. Several other salt bridges are also as frequently present as pS237–K240. In C36, pT231–R230 is the most occurring salt bridge, but both pS327–R242 and pS235–K234 are more probable than pS237–K240. Hence, while both force fields captured the experimentally established salt bridges, they also suggested other salt bridges to be present and some of them to be more common than the experimentally established ones.

**Figure 4:**
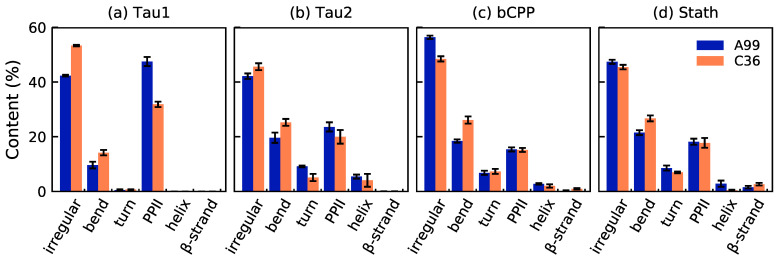
Average content of different types of the secondary structure in (**a**) Tau1, (**b**) Tau2, (**c**) bCPP, and (**d**) Stath simulated with Amber ff99SB-ILDN (A99) and CHARMM36m (C36). The legend applies to all panels. The helix includes the α- 3_10_- and a negligible content of the π-helix, while the β-strand also includes β-bridge. Error bars correspond to errors calculated by block averaging.

Advancing to the secondary structure, Figure 4 shows that the peptides were mainly irregular, although Tau1 contained much of the polyproline type II (PPII) structure as well. In fact, all peptides contained a significant amount of PPII, as well as a significant content of bends. The content of the helical structure (α- and 3_10_-helix) and β-strands was low in all peptides. Tau1 exhibited the largest differences between the force fields, where A99 produced 16 percentage points more of the PPII structure than C36, which instead contained a more irregular structure. For the other peptides, the differences were smaller. Overall, the peptides only had one significant difference in common, which was a higher content of bends in C36 than A99. Inspecting the content along the sequence, it was evident that it was mostly the same parts of the peptide that were enriched in a certain type of structure in both force fields (see Supplementary Figure S3). However, in C36, the helical content was completely missing from the first ten residues of Stath, which is concerning since the N-terminal region has been shown to possess helical propensity in water, although being mainly disordered [6,30]. Another striking difference between the force fields for Stath is that some residues centered on residues Y21 and Y41 occasionally formed a β-sheet or β-bridge in C36, but not in A99. Notice also that for Tau2, the bend propensity at residues V228–V229 was much higher in C36 than in A99. Since these residues were located right between K225 and pT231, which in C36 formed a stable salt bridge, this suggested that the bend was formed as a result of the salt bridge. Furthermore, for Tau2, NMR data have suggested approximately 40% α-helical propensity in region A15-R18 [12]. Both A99 and C36 sampled the helical structure in this region, however, to a lower extent than what the experimental data suggested.

### 2.3. Energy Landscapes

**Figure 5:**
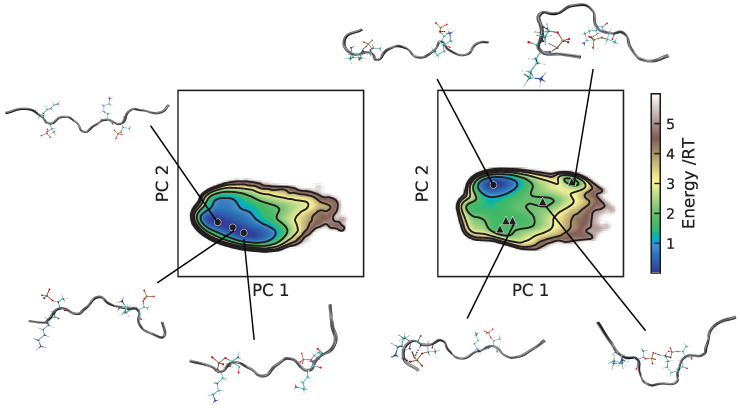
Energy landscapes and conformations in selected minima of Tau1. (**Left**) A99; (**right**) C36. The energy landscapes are constructed using the first two components from principal component analysis, using the same basis set for both force fields, such that they are directly comparable. Contour lines are drawn for integer energy levels in the interval 1 ≤ RT ≤ 5, and the minimum of each basin is represented by a marker: ●: energy ≤1RT, ▲: ≤2RT. In the conformations, the phosphorylated and positively charged residues are shown explicitly. Dashed black lines represent hydrogen bonds. The peptide conformations are color-coded according to the secondary structure determination in VMD, where silver is irregular (coil) and cyan is turns. The N-terminus of each conformation is the leftmost end.

**Figure 6:**
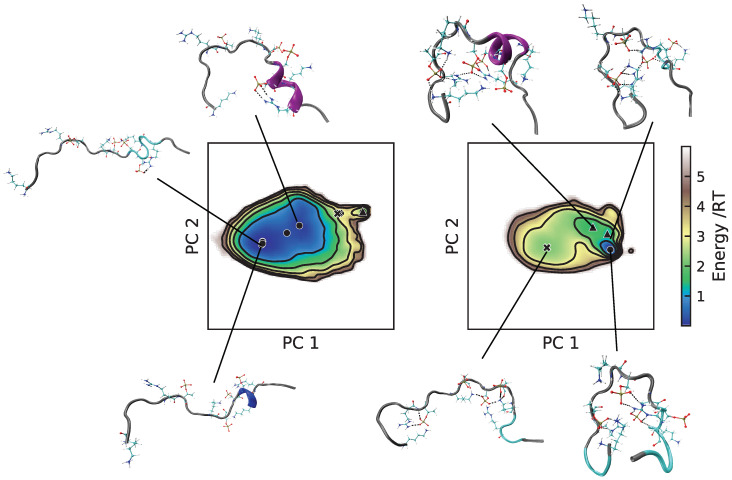
Energy landscapes and conformations in selected minima of Tau2. (**Left**) A99; (**right**) C36. The energy landscapes are constructed using the first two components from principal component analysis, using the same basis set for both force fields, such that they are directly comparable. Contour lines are drawn for integer energy levels in the interval 1 ≤ RT ≤ 5, and the minimum of each basin is represented by a marker: ●: energy ≤1RT, ▲: ≤2RT, ✖: ≤3RT. In the conformations, the phosphorylated and positively charged residues are shown explicitly. Dashed black lines represent hydrogen bonds. The peptide conformations are color-coded according to the secondary structure determination in VMD, where silver is irregular (coil), cyan is turns, magenta is the α-helix, and blue is the 3_10_-helix. The N-terminus of each conformation is the leftmost end.

**Figure 7:**
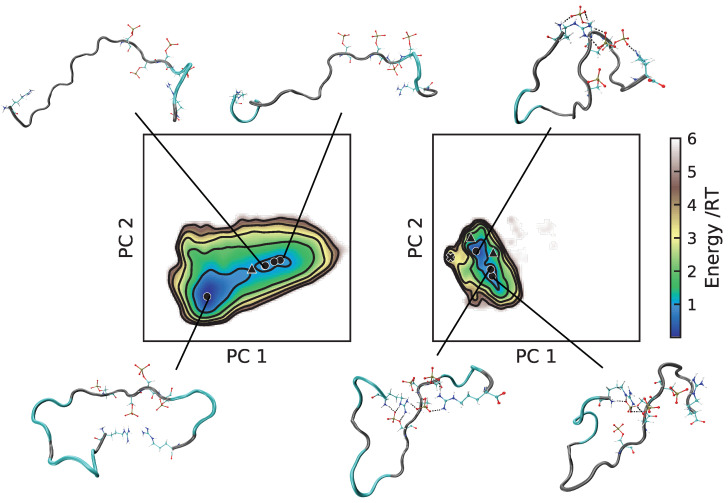
Energy landscapes and conformations in selected minima of bCPP. (**Left**) A99; (**right**) C36. The energy landscapes are constructed using the first two components from principal component analysis, using the same basis set for both force fields, such that they are directly comparable. Contour lines are drawn for integer energy levels in the interval 1 ≤ RT ≤ 5, and the minimum of each basin is represented by a marker: ●: energy ≤ 1RT, ▲: ≤2RT, ✖: ≤3RT. In the conformations, the phosphorylated and positively charged residues are shown explicitly. Dashed black lines represent hydrogen bonds. The peptide conformations are color-coded according to the secondary structure determination in VMD, where silver is irregular (coil) and cyan is turns. The N-terminus of each conformation is the leftmost end.

**Figure 8:**
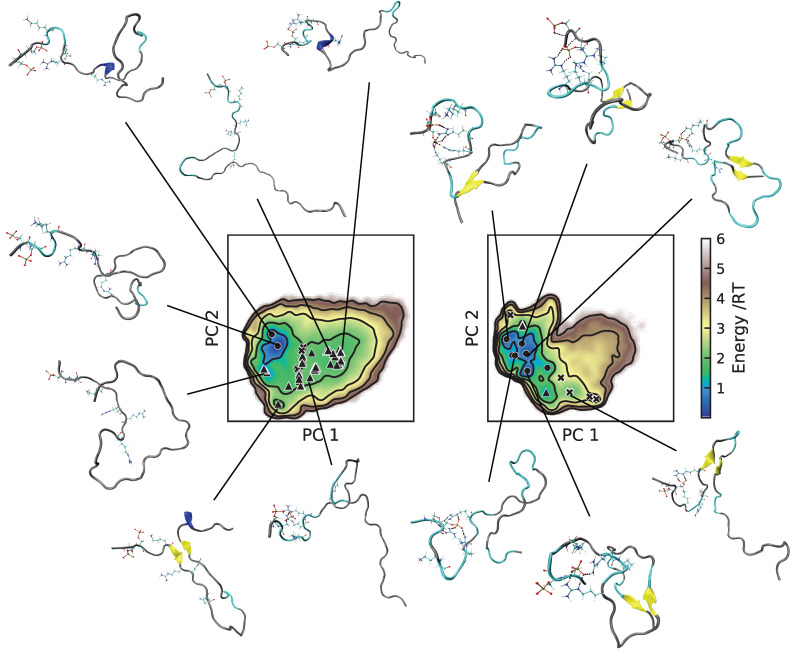
Energy landscapes and conformations in selected minima of Stath. (**Left**) A99; (**right**) C36. The energy landscapes are constructed using the first two components from principal component analysis, using the same basis set for both force fields, such that they are directly comparable. Contour lines are drawn for integer energy levels in the interval 1 ≤ RT ≤ 5, and the minimum of each basin is represented by a marker: ●: energy ≤ 1RT, ▲: ≤2RT, ✖: ≤3RT. In the conformations, the phosphorylated and positively charged residues are shown explicitly. Dashed black lines represent hydrogen bonds. The peptide conformations are color-coded according to the secondary structure determination in VMD, where silver is irregular (coil), cyan is turns, blue is the 3_10_-helix, yellow is the β-sheet, and tan is the β-bridge. The N-terminus of each conformation is the leftmost/topmost end.

The differences between the force fields in this study is well summarized by the energy landscapes in Figure 5, Figure 6, Figure 7 and Figure 8. Tau2, bCPP, and Stath all showed a narrower energy landscape in C36, in line with a more restricted conformational ensemble. Tau1, which is rather short and stiff, actually gained a larger conformational landscape in C36, due to sampling more bent conformations in addition to being more stretched out as in A99; see Figure 5. Notice also that in C36, the global minimum, which was the most populated, contained conformations that were not entirely stretched out. Instead, the N-terminal end was folded over, such that a salt bridge was formed between pT175 and the positively charged N-terminus.

Although the energy landscapes of Tau2 in A99 and C36 were located in almost the same area, the energy levels differed; see Figure 6. The most populated basin in the C36 simulation was a deep and narrow minimum, while the A99 simulation had a larger area of energy ≤1RT, containing several basins, more typical of IDPs. The salt bridges creating more compact conformations were evident in the C36 conformations, while the A99 conformations were more stretched out with fewer salt bridges. Notice that the phosphorylated residues in C36 had a tendency to interact with several positively charged residues simultaneously. In both force fields, a basin minimum with a helical region starting with pS237 and pS238 was found, in line with the secondary structure analysis.

For bCPP, there was indeed many more elongated conformations in the A99 simulation (see Figure 7), and it is clear that what caused the more compact conformations in C36 was the salt bridges between the phosphorylated serines and the arginines. In C36, all depicted conformations contained at least one salt bridge between phosphoserine and arginine, while this was much rarer in A99, explaining why the energy landscapes looked so different. Regarding Stath, comparing the conformations in Figure 8, there were two striking differences. First, there was a higher presence of salt bridges between phosphoserine and positively charged residues in C36, keeping the N-terminal end in a more bent conformation. Secondly, in C36, the β-strand and β-bridge formation between the middle region and C-terminal region detected in Supplementary Figure S3 contributed to making the conformations more compact compared to A99.

### 2.4. Effect of Salt Concentration

**Figure 9:**
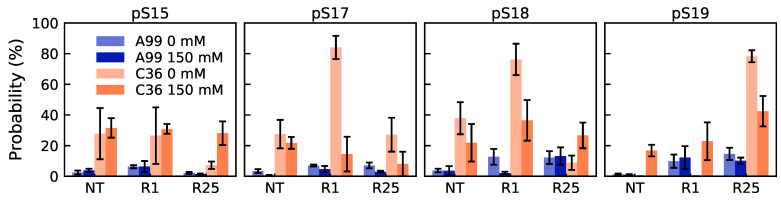
Probability of possible salt bridge interactions for the phosphorylated residues with the N-terminus (NT) and positively charged residues in bCPP, simulated with the two different force fields in the presence of 0 mM or 150 mM NaCl. Error bars corresponds to errors calculated by block averaging.

Since the salt bridges formed between phosphorylated and positively charged residues were shown to influence the conformational ensemble, it is of importance to also consider the effect of the screening of the electrostatic interactions. Here, we focused on bCPP, which due to showing the largest differences between force fields and having the highest fraction of charged residues in combination with the largest charge separation (see Supplementary Table S1), was expected to show the largest response to ionic strength. Figure 9 shows that in C36, four of the salt bridges were dramatically reduced upon the addition of 150 mM NaCl; however, the probability of two other salt bridges increased, whereas in A99, only one salt bridge was significantly reduced. At 150 mM salt, the salt-bridging probability was more comparable between A99 and C36, although overall still higher in C36. Supplementary Figure S3 shows the changes in the contact map upon the addition of 150 mM NaCl for bCPP simulated in A99 and C36. For A99, we clearly saw that the preference for the N-terminal end to be in contact with the phosphorylated and negatively charged region (residues 14–21) diminished. In C36, the strongly conserved R1–pS17 and R1–pS18 contacts were greatly decreased, while the contact of R1 with surrounding residues in the negatively charged region was increased. Hence, this suggested an increased mobility, while still maintaining contact with the negatively charged region. In C36, the cross-diagonal lines also signalized a decrease of the β-sheet; however, the content was relatively low from the beginning.

**Figure 10:**
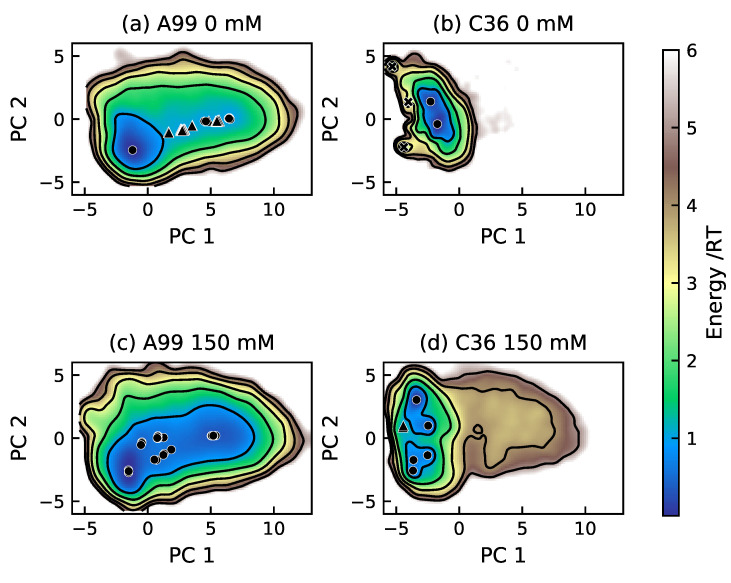
Energy landscapes of bCPP simulated with the two force fields Amber ff99SB-ILDN (A99) and CHARMM36m (C36) in the presence of 0 mM or 150 mM NaCl.

By comparing the energy landscapes in Figure 10, it is clear that screening of the electrostatic interactions indeed broadened the conformational ensemble, but mainly in C36, which also showed the largest change in salt bridge probability. In C36, the addition of 150 mM NaCl led to the exploration of more stretched out conformations; however, more compact conformations still clearly dominated. A99 also showed an increased probability of visiting more stretched out conformations after the addition of 150 mM NaCl. This shift in the conformational ensemble was also observed in the distributions of R_g_ and R_ee_ shown in Supplementary Figure S4. However, the changes were actually rather small, such that the average values were indistinguishable. Upon the addition of salt, the R_g_ changed from 1.43±0.03 nm to 1.45±0.03 nm for A99 and from 1.08±0.02 nm to 1.08±0.03 nm for C36. The changes in R_ee_ were from 3.09±0.15 nm to 3.37±0.13 nm and from 1.65±0.10 nm to 1.67±0.10 nm, respectively. The effect of salt on the calculated scattering curves was also so small that it could be deemed negligible; see Supplementary Figure S5.

## 3. Conclusions

C36 produced more compact conformations of all four peptides, which indeed was expected to be caused mainly by salt bridge stability. In Tau1, the salt bridges pT175–K174 and pT181-K180 were formed without much effect on the overall conformation; however, an additional salt bridge between the N-terminus and pT175 decreased R_ee_ and R_g_ in C36. In Stath, the salt bridges contributed to the discrepancy by restricting the conformation of the first 15 residues, in the same way as previously shown for that fragment studied alone [20]. However, also the β-bridge and β-strand formation between the middle and C-terminal region were shown to contribute to more compact conformations. While C36 produced good results of nonphosphorylated short IDPs, it has been shown to underestimate the size of larger IDPs (>60 residues) [31,32]. Since Stath was 43 residues long, and thus the longest peptide included in this study, it is reasonable to believe that other effects also play a role. That bCPP showed the largest difference between the force fields and Tau1 the smallest implies that the separation between the phosphorylated and positively charged residues controls how much the conformational ensemble is influenced by stable salt bridges. This is in accordance with the importance of considering the level of charge separation for predicting the conformational ensemble of IDPs with a high fraction of charges [33].

When comparing to experimental data, it is important to consider the effect of salt, since most experiments are performed in the presence of buffer and additional salt. In bCPP, the addition of 150 mM NaCl was shown to dramatically reduce the probability of some of the salt bridges in C36, whereas the probability of other salt bridges actually increased. In A99, only one salt bridge was significantly reduced, which suggests that salt bridges still are of importance at 150 mM NaCl. Considering the changes in salt bridge probability for bCPP with salt concentration, it is plausible that the discrepancies between the simulations and experimental reference for Tau2 were caused by nonmatching ionic strength, since the experiments were performed with 50 mM phosphate buffer. At the same time, it can be hard to discern the salt bridges involving close-by residues experimentally, such as for pS237, pS238, K240, and R242.

Despite significant differences in the salt-bridging probability in C36, the effect of salt concentration on the global conformational level, such as R_g_ and R_ee_, was small enough to be negligible for both force fields. In fact, the calculated form factor was indistinguishable, implying that comparing simulations performed without salt with experimental SAXS data collected at 150 mM NaCl indeed can be valid. Since bCPP is the peptide for which we expected the largest effects of salt concentration, this further strengthens the comparison with SAXS data for Stath collected at 150 mM NaCl, which showed that A99 was in good agreement, while C36 overestimated the level of compaction. Although the effects of ionic strength seem negligible in this study, this is generally not the case. For example, Jin and Gräter needed 350 mM of salt in simulations with A99 to reach experimental agreement for IDPs that are approximately 80 residues long [21], which suggested that also A99 overestimate the strength of salt bridges. Here, both Tau1 and Stath were compared to experimental size estimates, and only C36 was with certainty shown to underestimate the size. Hence, a possible overestimation of salt bridge stability in A99 is not expected to be a major issue for describing the conformational ensemble of the short IDPs studied in this work. This emphasizes the importance of benchmarking against IDPs of different length and sequence when developing and evaluating force fields. While a reduction of the strength of salt bridges appears to be a crucial step in improving the performance of C36, it appears less critical in A99. However, note that this statement is based only on the global conformational properties and that it might be different for studies of dynamics. Based on observations that many force fields have a tendency to overstabilize salt bridges, which seems to be related to side-chain partial charges [22,34,35,36], we suggest that readjusting the side-chains’ partial charges, especially of the phosphorylated residues, is a way of improving the force fields.

Another area which has not been touched upon in this work is the influence of charge regulation and pH. The simulations have been performed with fixed charges in a state corresponding to physiological pH, where the phosphorylated residues have have a charge of −2e. Since the pKa of the phosphorylated residues is around six [37], in reality it can fluctuate between −1e and −2e. Recent studies have suggested the importance of the protonation state of phosphorylated residues for molecular interactions [38], hence influencing salt bridge formation and the conformational ensemble. Therefore, this is suggested to be included in future investigations.

Considering the secondary structure, the only general difference between the force fields was a higher content of bends in C36. In Tau2, it was focused on regions between salt-bridging-forming partners, suggesting that highly stable salt bridges can enforce bends depending on the separation between the salt-bridging residues. For Tau2, it was suggested that both force fields underestimated the helical propensity, and in Stath, a lack of helix propensity in the N-terminal regions was concerning for C36. However, to properly assess the performance of force fields regarding the secondary structure, detailed experimental references are important. Hence, we see that NMR experiments of phosphorylated IDPs recording coupling constants, NOEs, and chemical shifts, which capture the effects of both the secondary structure and salt bridges, are an essential part of improving force fields. Since atomistic simulations can be used to carefully detect the secondary structure and salt bridges and their dynamics, it is an important tool in understanding the mechanism behind the regulation of IDP function by phosphorylation, provided that sufficient accuracy of the force fields is achieved.

## 4. Materials and Methods

Fraction of charged residues and ϰ, a parameter describing how segregated the charged residues are in the sequence [33] were calculated in CIDER [39], by equalizing the phosphorylated residues to other negatively charged residues. The value of ϰ is normalized against the most segregated sequence for that sequence composition, therefore adopting a value in the range 0–1, where 1 corresponds to the most segregated sequence possible.

The simulations listed in Supplementary Table S3 were performed in GROMACS 2018.4 [40,41,42,43,44], using two different force fields: Amber ff99SB-ILDN [23] with the TIP4P-D [24] water model and parameters for the phosphorylated residues from Homeyer et al. [25] and Steinbrecher et al. [26], and CHARMM36m [27] with the CHARMM-modified TIP3P water model [28]. Initial configurations of the peptides were constructed from the sequence as linear chains using Avogadro 1.2.0 [45], optimizing the structure with the auto-optimization tool. Each peptide was solvated in a rhombic dodecahedron box, having a minimum distance between the peptide and the box edges of 1 nm. Sodium ions were added to neutralize the system, and two systems were also simulated with sodium and chloride ions in a concentration corresponding to 150 mM. Periodic boundary conditions were employed in all directions. The equations of motion were integrated using the Verlet leapfrog algorithm [46] with a time step of 2 fs. Nonbonded interactions were treated with a Verlet list cutoff scheme. The short-range interactions were calculated using neighbor lists with cutoff 1 nm or 1.2 nm, for the Amber and CHARMM force fields, respectively. For the CHARMM force field, the Lennard–Jones interactions were switched off smoothly (force-switch) between 1 nm and 1.2 nm. Long-range dispersion corrections were applied to energy and pressure in the case of the Amber force field. Long-range electrostatic interactions were treated by particle mesh Ewald [47] with a cubic interpolation and a 1.6 Å grid spacing. The LINCS algorithm [48] was used to constrain all bond lengths in the case of Amber and only bonds with hydrogen atoms in the case of CHARMM. The solute and solvent were separately coupled to temperature baths at 298 K using the velocity rescaling thermostat [49] with a 0.1 ps relaxation time. Parrinello–Raman pressure coupling [50] was used to keep the pressure at 1 bar, using a 2 ps relaxation time and $4.5\cdot 10^{-5}$ bar^−1^ isothermal compressibility.

Energy minimization was performed by the steepest descent algorithm until the system converged within the available machine precision. Initiation of five replicates per system with different starting seeds was performed separately in two steps using position restraints on the peptide. The first step was 500 ps of NVT simulation (constant number of particles, volume, and temperature) performed to stabilize the temperature, followed by the second step of 1000 ps of NPT simulation (constant number of particles, pressure, and temperature) to stabilize the pressure. Production runs of the five replicates per system were performed in the NPT ensemble, for at least 1 µs per replicate. The total simulation time per system is stated in Supplementary Table S3. Energies and coordinates were saved every 10 ps. Supplementary Tables S4 and S5 compile a few differences applied to the salt simulations to reduce the computational time.

### Analysis

The convergence and sampling quality were assessed in the following ways. The time evolution of the R_g_ and the R_ee_ in the simulations were observed for signs of equilibration in the initial stage. Based on this, the first 30 ns were removed from each replicate of bCPP in CHARMM36m and the first 50 ns of each replicate of Tau2 in CHARMM36m before final analysis (see Supplementary Figures S21 and S24). In other systems the equilibration was deemed fast enough to be negligible. The distributions of the R_g_ and the R_ee_ as well as the energy landscapes were compared between replicates, since similarity indicates sufficient sampling. The autocorrelation function and block average error estimates of the R_g_ and the R_ee_ in the concatenated simulation were calculated and observed for an estimate of the correlation time and convergence of the error estimates. All this data is presented in the Supplementary Figures S6–S33. Although some systems showed greater dissimilarity between replicates than desired, based on the assessment of the concatenated trajectory, it was deemed sufficiently sampled to allow for a comparison between the force fields.

R_g_ and R_ee_ were calculated using GROMACS 2018.4 [40,41,42,43,44]. Reported error estimates were calculated using block averaging analysis as implemented in the *gmx analyze* routine in GROMACS. Scattering curves were calculated using CRYSOL Version 2.8.3 [51] with the contrast of the hydration shell being 0.0075 *e*/Å^3^ for Amber ff99SB-ILDN+TIP4P-D and 0.02 *e*/Å^3^ for CHARMM36m, as suggested by [29]. The presented curve is the average over 10,000 equally spaced frames. In Supplementary Figure S1 and Table S2, the effect of different contrasts of the hydration shell is shown for Stath. The quality of fit to the experimental curve is computed as:

(1) $$\chi^{2}\left(f,c\right)=N_{q}^{-1}\sum_{i=1}^{N_{q}}{\left[\frac{I_{\mathrm{ref}}\left(q_{i}\right)-\left(fI_{\mathrm{obs}}\left(q_{i}\right)+c\right)}{\sigma_{\mathrm{ref}}\left(q_{i}\right)}\right]}^{2},$$

where $N_{q}^{-1}$ is the number of points in the reference curve, $I_{\mathrm{ref}}$ and $I_{\mathrm{obs}}$ are the reference and observed intensities, respectively, and $\sigma_{\mathrm{ref}}\left(q_{i}\right)$ is the error associated with each data point of the reference curve. The function was minimized using the Nelder–Mead method [52], as implemented in Scipy [53], using linear interpolation to produce $I_{\mathrm{obs}}$ at the same *q* points as the reference [29]. AUTORG in the ATSAS program [54] was used to determine the R_g_ from Guinier analysis. The secondary structure was determined using the DSSP program Version 2.2.1 [55] with an extension to detect the polyproline type II structure [56,57]. The MDTraj Python library Version 1.9.3 [58] was used to calculate contact probability and analyze salt bridges. Contact between two residues was defined as when the shortest distance between two atoms <0.4 nm. Since salt bridges are formed as a result of hydrogen bonding and electrostatic interactions, they were assessed by analyzing the presence of hydrogen bonds based on the criterion in [59], as implemented in MDTraj. Energy landscapes were calculated following the Campos and Baptista approach [60], with the differences described by Henriques et al. [61]. In short, principal component analysis was applied to the Cartesian coordinates of the backbone atoms of the protein, obtained after translational and rotational least squares fitting on the central structure of the simulation. The conditional free energy was calculated from the probability density function in the representation space constructed by the first two principal components, obtained by Gaussian kernel density estimation. The basins and minima were assigned as described by Campos and Baptista [60]. It is worth noting that the first two components were shown to account for 46–60% of the variance, hence not providing a complete picture of the conformational classes, but at least an overview sufficient for comparison between the force fields. Snapshots from the simulations were produced using VMD 1.9.3 [62,63,64].

## Figures and Tables

**Table 1:** Full name and sequence of the peptides included in this study. Positively charged residues are marked in blue, negatively charged in red, and phosphorylated residues highlighted with yellow. Note that Tau1 includes three additional residues in accordance with [11], to allow for experimental comparison.

Name	Protein	Sequence
Tau1	Tau_173-183_	CAKTPPAPKTPPAW
Tau2	Tau_225-246_	KVAVVRTPPKSPSSAKSRLQTA
bCPP	β-casein_1-25_	RELEELNVPGEIVESLSSSEESITR
Stath	Statherin	DSSEEKFLRRIGRFGYGYGPYQPVPEQPLYPQPYQPQYQQYTF

**Table 2:** Average radius of gyration and end-to-end distance of the peptides simulated with Amber ff99-SB-ILDN (A99) and CHARMM36m (C36). The difference between the force fields is expressed in relation to A99.

Peptide	Radius of Gyration (nm)			End-to-End Distance (nm)		
	**A99**	**C36**	**Difference (%)**	**A99**	**C36**	**Difference (%)**
Tau1	1.17±0.01	1.12±0.01	4	3.44±0.04	2.88±0.07	16
Tau2	1.29±0.03	1.06±0.10	18	3.27±0.17	2.10±0.32	36
bCPP	1.43±0.03	1.08±0.02	24	3.09±0.15	1.65±0.10	47
Stath	1.73±0.09	1.41±0.04	18	4.05±0.17	2.74±0.20	32

## Data Availability

Data is included in the article or Supplementary Materials.

## Supplementary Materials

The following are available online at https://www.mdpi.com/article/10.3390/ijms221810174/s1.

## Abbreviations

A99	Amber ff99SB-ILDN with TIP4P-D water
C36	CHARMM36m with CHARMM-modified TIP3P water
FRET	Fluorescence resonance energy transfer
IDP	Intrinsically disordered protein
NMR	Nuclear magnetic resonance
PPII	polyproline type II
R_g_	Radius of gyration
R_ee_	End-to-end distance
SAXS	Small-angle X-ray scattering
