# Supplementary material for: Molecular Dynamics Simulations of Phosphorylated Intrinsically Disordered Proteins: A Force Field Comparison
Source: Int J Mol Sci. 2021 Sep 21;22(18):10174. doi: 10.3390/ijms221810174 (PMC8470740; doi:10.3390/ijms221810174)
Supplement: Supplementary file 1 [file ijms-22-10174-s001.zip › ijms-1384558-supplementary 2.pdf]

Supplementary Materials for Molecular Dynamics Simulations of Phosphorylated Intrinsically Disordered Proteins: A Force Field Comparison

E. Rieloff and M. Skepö

This file contains:

- Table S1: Fraction of charged residues and $\varkappa$ of the peptides studied.
- Table S2: Radius of gyration and χ^2 of calculated scattering curves using different contrast of hydration shell ($\delta\rho$) for Stath.
- Table S3: Details of the simulations performed in this work.
- Table S4: Differences in the setup of systems with 150 mM NaCl.
- Table S5: Starting configuration used in simulations of system bCPP 150 mM C36.
- Figure S1: Calculated scattering curves of Stath using different contrast of the hydration shell.
- Figure S2: Content of different secondary structure elements along the sequence for the four peptides.
- Figure S3: Difference in contact probability for bCPP between 0 and 150 mM NaCl.
- Figure S4: Distribution of radius of gyration of end-to-end distance for bCPP with 0 and 150 mM NaCl.
- Figure S5: Calculated scattering curves for bCPP with 0 and 150 mM NaCl.
- Figure S6-S8: Plots for assessing convergence and sampling quality of the simulations of Tau1 in Amber ff99SB-ILDN.
- Figure S9-S11: Plots for assessing convergence and sampling quality of the simulations of Tau2 in Amber ff99SB-ILDN.
- Figure S12-S14: Plots for assessing convergence and sampling quality of the simulations of bCPP in Amber ff99SB-ILDN.
- Figure S15-S17: Plots for assessing convergence and sampling quality of the simulations of Stath in Amber ff99SB-ILDN.
- Figure S18-S20: Plots for assessing convergence and sampling quality of the simulations of Tau1 in CHARMM36m.
- Figure S21-S23: Plots for assessing convergence and sampling quality of the simulations of Tau2 in CHARMM36m.

- Figure S24-S26: Plots for assessing convergence and sampling quality of the simulations of bCPP in CHARMM36m.
- Figure S27-S29: Plots for assessing convergence and sampling quality of the simulations of Stath in CHARMM36m.
- Figure S30-S31: Plots for assessing convergence and sampling quality of the simulations of bCPP with 150 mM NaCl in Amber ff99SB-ILDN.
- Figure S32-S33: Plots for assessing convergence and sampling quality of the simulations of bCPP with 150 mM NaCl in CHARMM36m.

Table S1: Fraction of charged residues (FCR) and level of charge separation described by $\varkappa$ of the peptides studied.

Peptide	FCR	$\varkappa$
Tau1	0.29	0.05
Tau2	0.41	0.12
bCPP	0.52	0.46
Stath	0.23	0.32

Table S2: Radius of gyration and χ^2 of calculated scattering curves using different contrast of hydration shell ($\delta\rho$) for Stath simulated with Amber FF99-SB-ILDN (A99) and CHARMM36m (C36). The R_g is obtained from Guinier analysis using AUTORG in the ATSAS program [1], and the error reported is the estimated error given by AUTORG.

	**A99**		**C36**	
$\delta\rho(e/Å^3)$	R_g (Å)	χ^2	R_g (Å)	χ^2
0	17.2 ± 0.6	2.0	13.9 ± 0.4	6.7
0.01	17.5 ± 0.6	1.6	14.2 ± 0.3	5.3
0.02	17.8 ± 0.5	1.4	14.6 ± 0.3	4.4
0.03	18.1 ± 0.5	1.3	14.9 ± 0.3	3.7

Table S3: Details of the simulations performed in this work.

Peptide	Force field	Salt concentration (mM)	Box volume (nm^3)	Number of solvent molecules	Number of sodium ions	Number of chloride ions	Total simulation length (µs)
Tau1	A99	0	263.66	8637	2	0	5
Tau2	A99	0	722.941	23816	3	0	11
bCPP	A99	0	1002.41	32815	13	0	6
Stath	A99	0	942.11	30942	4	0	12
Tau1	C36	0	263.75	8495	2	0	11
Tau2	C36	0	722.93	23519	3	0	8.05
bCPP	C36	0	1002.48	32381	13	0	6.75
Stath	C36	0	950.87	30708	4	0	6
bCPP	A99	150	1002.41	32633	104	91	7
bCPP	C36	150	1002.48	32199	104	91	9.49

*A99 = Amber ff99SB-ILDN with the TIP4P-D water model, C36 = CHARMM36m with the CHARMM-modified TIP3P water model.

Table S4: Differences in the setup of the systems with 150 mM NaCl.

System	Number of replicates	Minimum simulation length of replicate (µs)	Saving frequency (pS)
bCPP 150 mM A99	10	0.7	40
bCPP 150 mM C36	10	0.48	50

Table S5: Starting configuration used in simulations of system bCPP 150 mM C36.

Replicate number	Starting configuration
1	Linear
2	Linear
3	Linear
4	Linear
5	Linear
6	t=58 ns in replicate #1
7	t=58 ns in replicate #2
8	t=58 ns in replicate #3
9	t=58 ns in replicate #4
10	t=58 ns in replicate #5

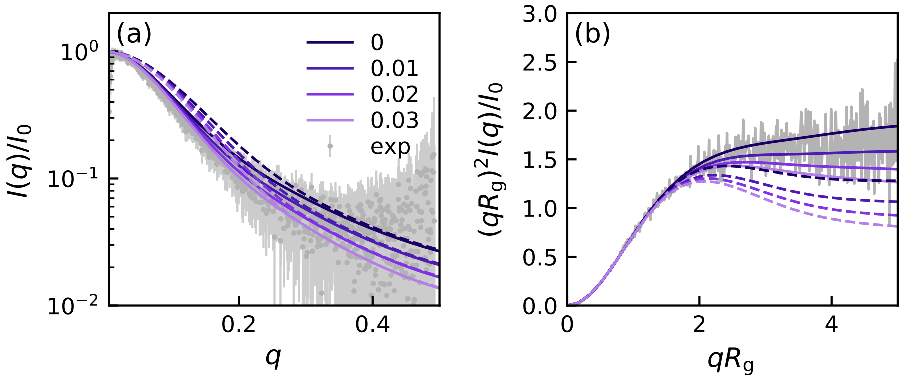

Figure S1: Calculated scattering curves of Stath using different contrast of the hydration shell presented as a semi-log plot (a) and dimensionless Kratky plot (b). Solid lines correspond to Amber ff99SB-ILDN+TIP4P-D and dashed lines to CHARMM36m. The experimental curve is the form factor of Statherin first presented in reference [2].

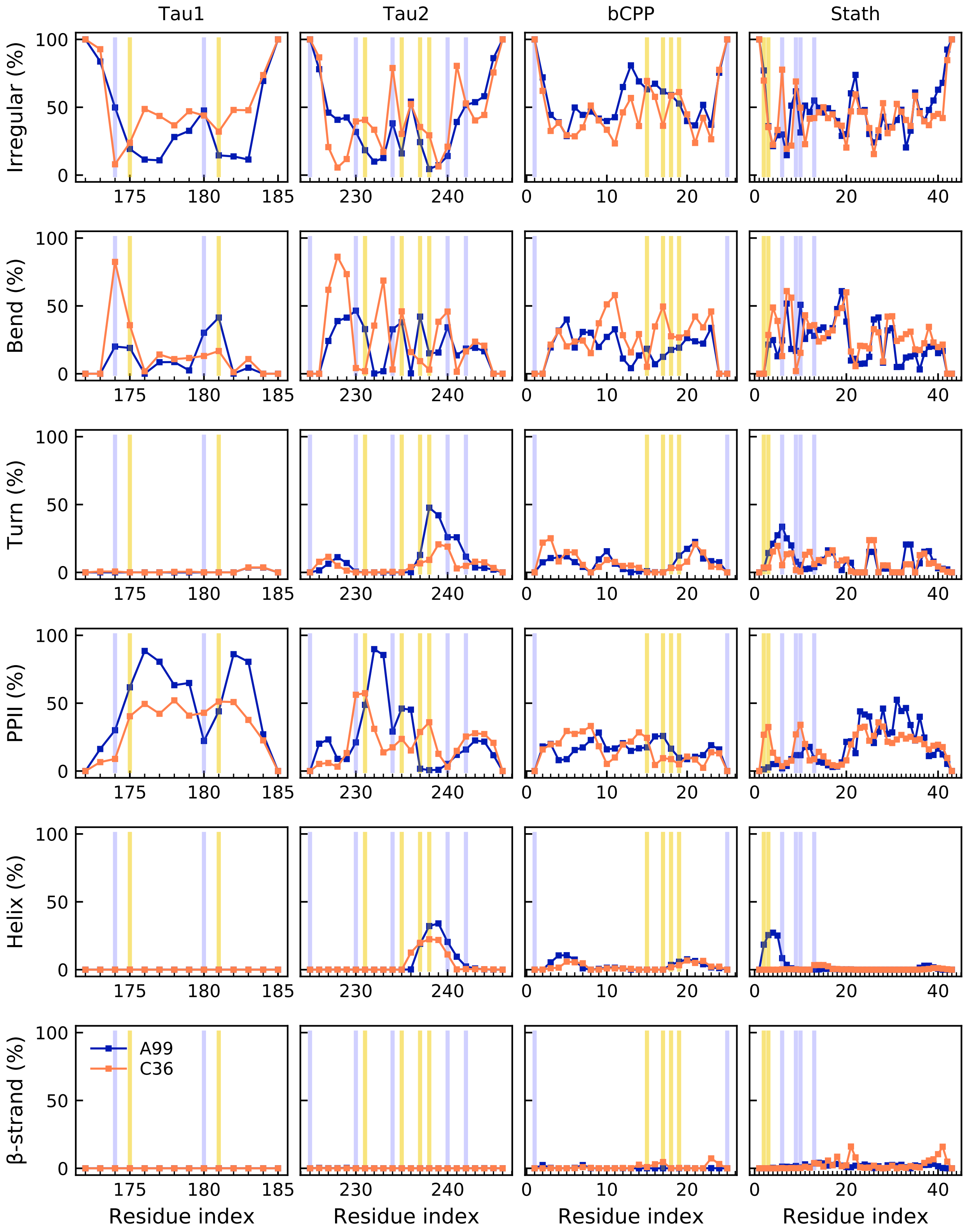

Figure S2: Content of different secondary structure elements along the sequence for the four peptides. The legend in the lower left panel applies to all panels. The position of phosphorylated residues are highlighted in yellow, and the position of positively charged residues in blue. Helix contains both α-, 3_{10}- and π-helix, and β-strand contain also includes β-bridge.

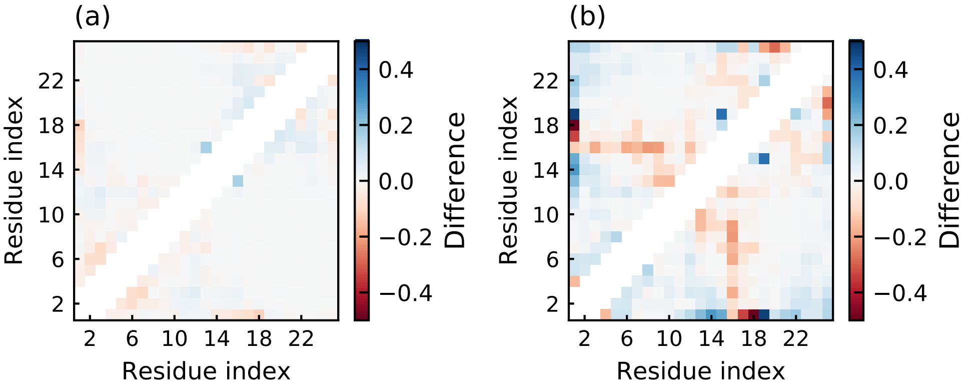

Figure S3: Difference in contact probability between 0 and 150 mM salt for bCPP simulated with Amber ff99SB-ILDN (a) and CHARMM36m (b).

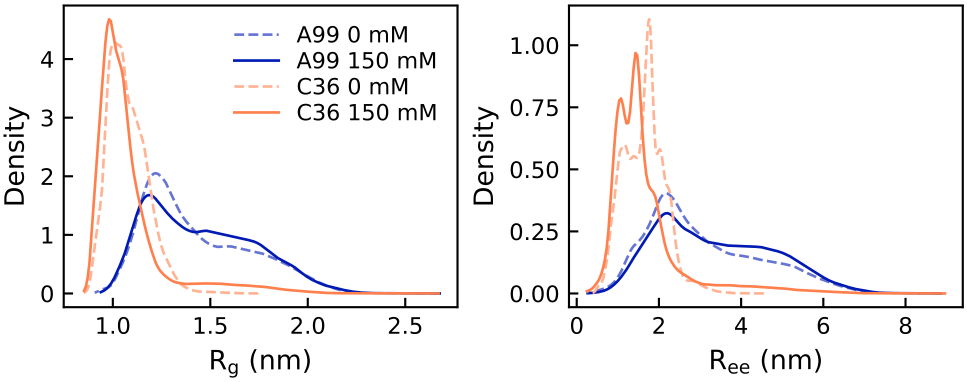

Figure S4: Distribution of radius of gyration (a) and end-to-end distance (b) of bCPP simulated with Amber ff99SB-ILDN (A99) and CHARMM36m (C36) in the presence of 0 or 150 mM NaCl. The legend applies to both panels.

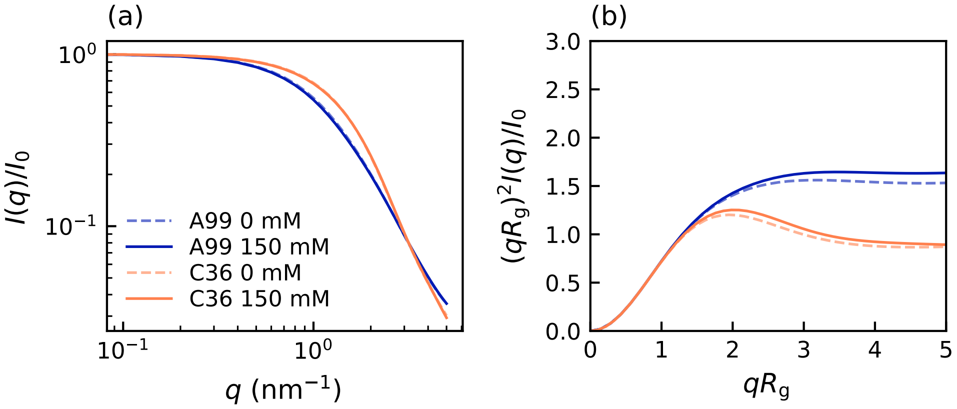

Figure S5: Calculated form factor (a) and dimensionless Kratky representation (b) of bCPP simulated with Amber ff99SB-ILDN (A99) and CHARMM36m (C36) in the presence of 0 or 150 mM NaCl. The legend applies to both panels.

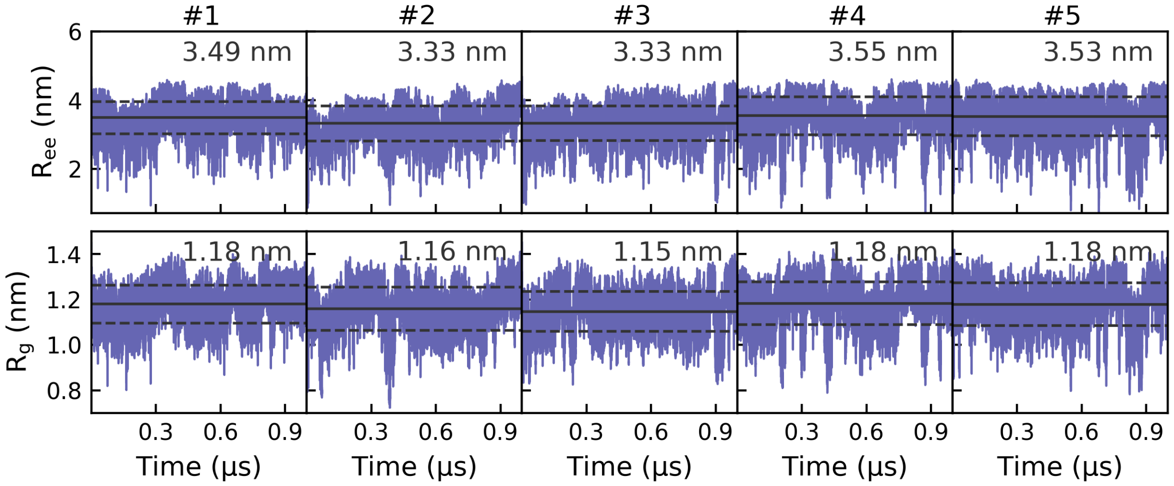

Figure S6: Time evolution of the end-to-end distance (R_{ee}) and the radius of gyration (R_g) for the five replicates in the simulation of Tau1 in Amber ff99SB-ILDN. The horizontal solid line represents the average in each replicate, with the dashed lines showing the standard deviation.

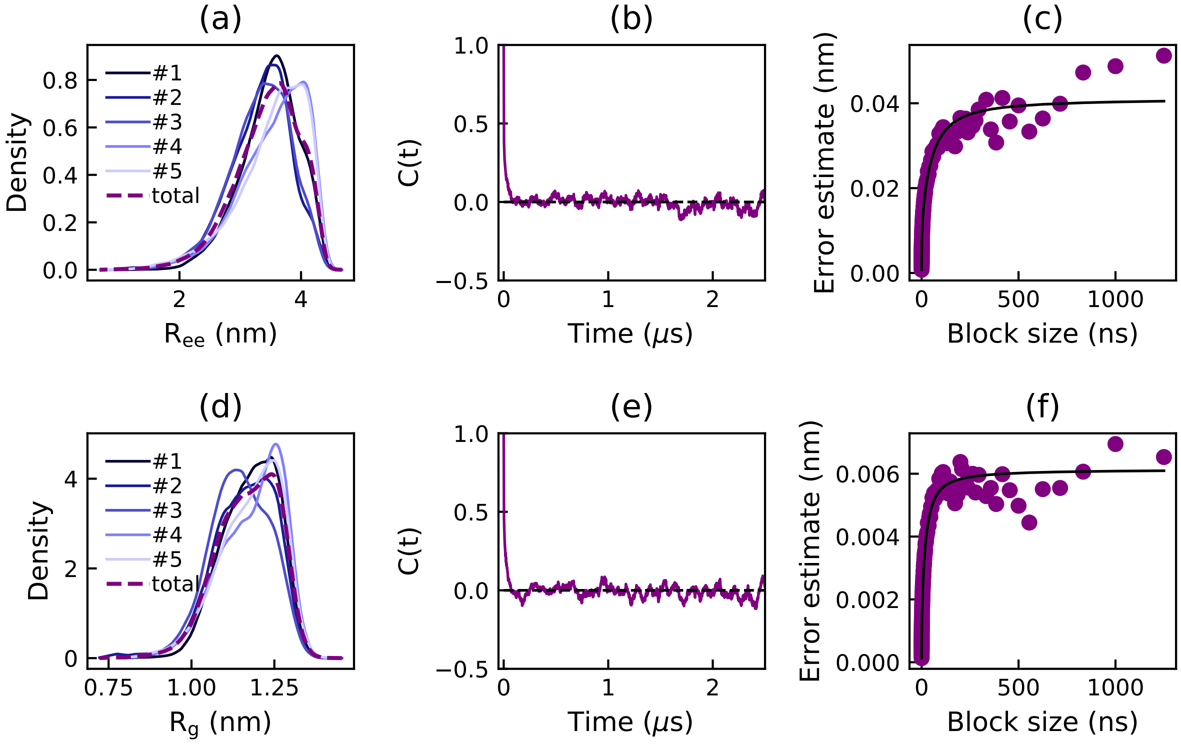

Figure S7: Density estimates of the end-to-end distance (R_{ee}) (a) and the radius of gyration (R_g) (d) for the five replicates and the concatenated simulation of Tau1 in Amber ff99SB-ILDN, obtained from a Gaussian kernel estimator. Autocorrelation function (C(t)) and error estimate from block averaging of the end-to-end distance (b,c) and the radius of gyration (e,f) for the concatenated simulation.

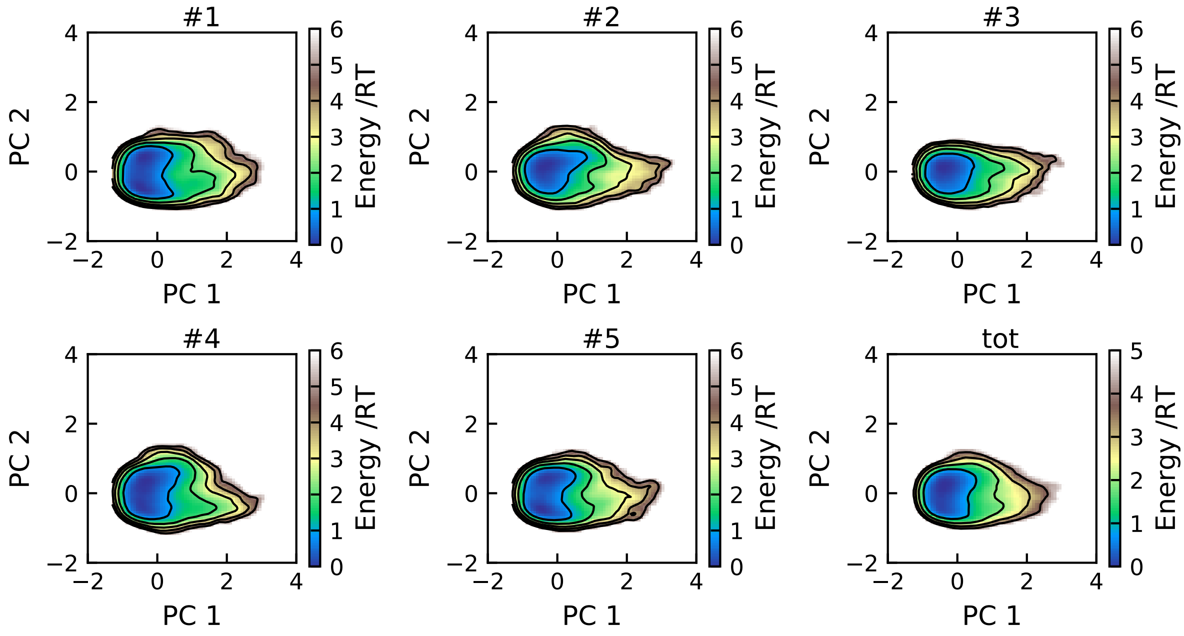

Figure S8: Energy landscapes for the five replicates and the concatenated trajectory of Tau1 in Amber ff99SB-ILDN, using the first two principal components. All plots have been constructed using the same basis set and are therefore directly comparable. Contour lines are drawn for integer energy levels in the interval $1 \leq RT \leq 5$.

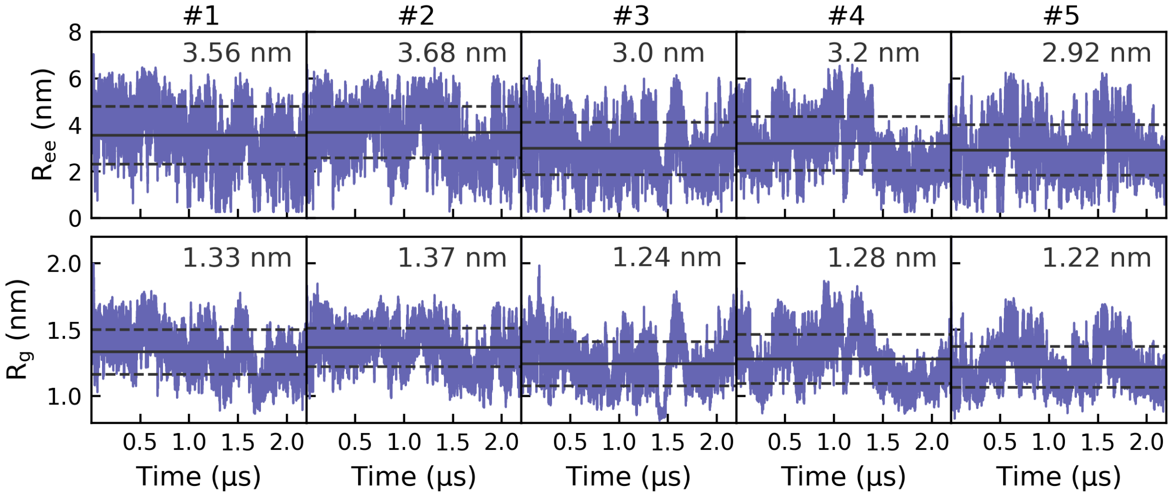

Figure S9: Time evolution of the end-to-end distance (R_{ee}) and the radius of gyration (R_g) for the five replicates in the simulation of Tau2 in Amber ff99SB-ILDN. The horizontal solid line represents the average in each replicate, with the dashed lines showing the standard deviation.

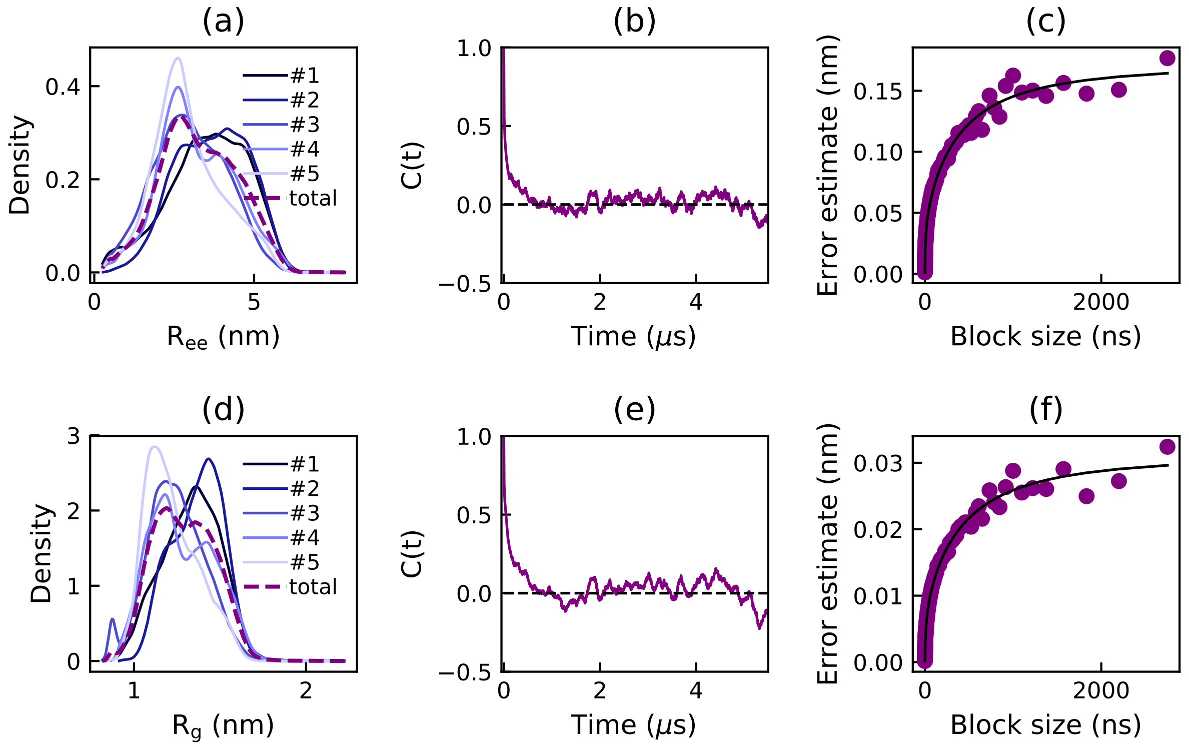

Figure S10: Density estimates of the end-to-end distance (R_{ee}) (a) and the radius of gyration (R_g) (d) for the five replicates and the concatenated simulation of Tau2 in Amber ff99SB-ILDN, obtained from a Gaussian kernel estimator. Autocorrelation function (C(t)) and error estimate from block averaging of the end-to-end distance (b,c) and the radius of gyration (e,f) for the concatenated simulation.

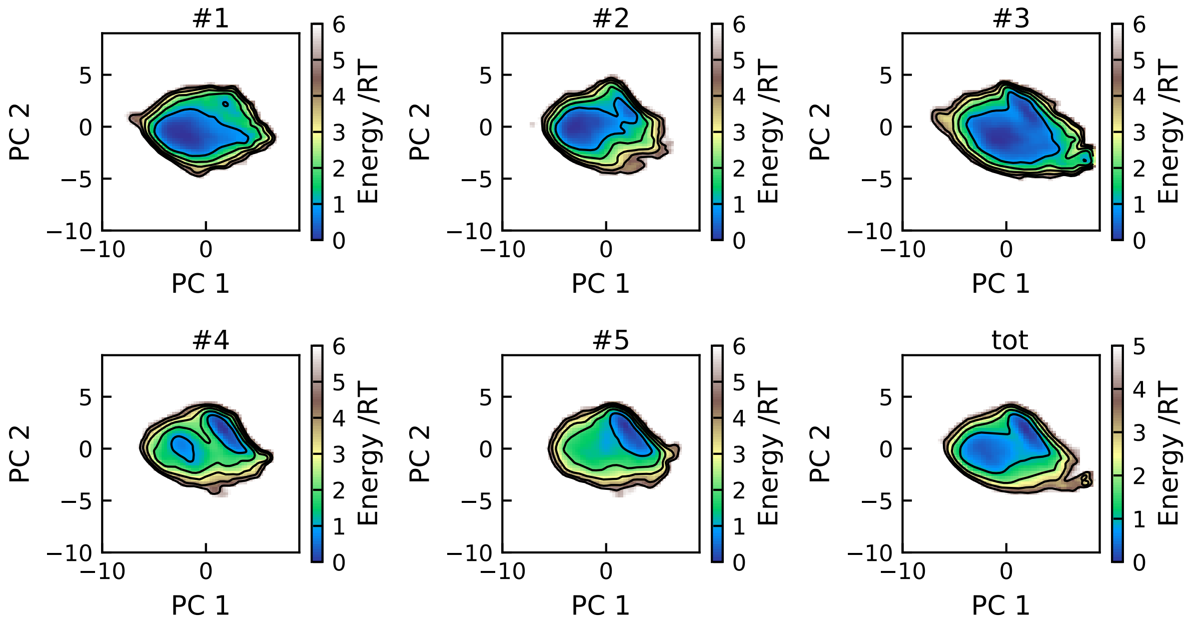

Figure S11: Energy landscapes for the five replicates and the concatenated trajectory of Tau2 in Amber ff99SB-ILDN, using the first two principal components. All plots have been constructed using the same basis set and are therefore directly comparable. Contour lines are drawn for integer energy levels in the interval $1 \leq RT \leq 5$.

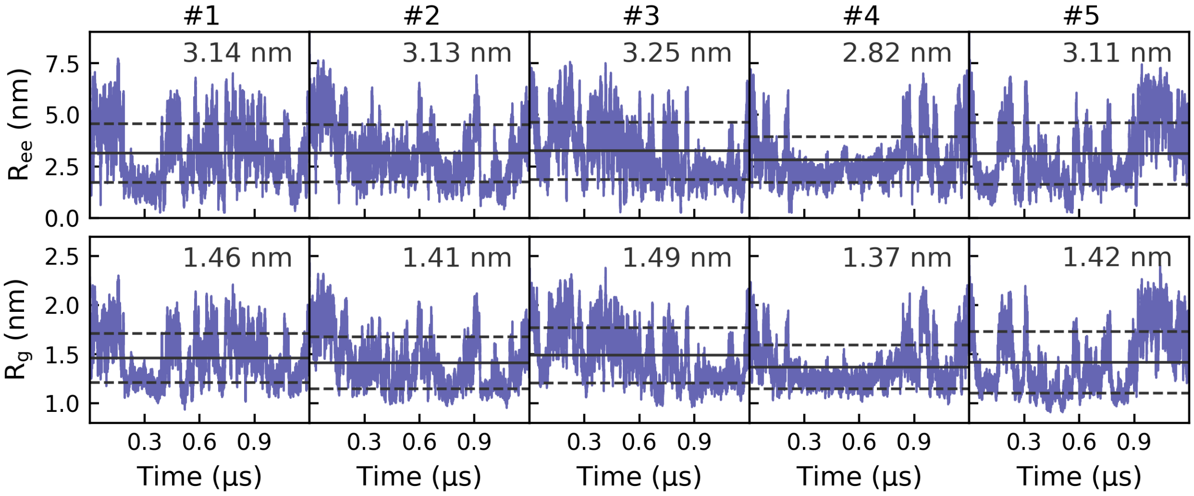

Figure S12: Time evolution of the end-to-end distance (R_{ee}) and the radius of gyration (R_g) for the five replicates in the simulation of bCPP in Amber ff99SB-ILDN. The horizontal solid line represents the average in each replicate, with the dashed lines showing the standard deviation.

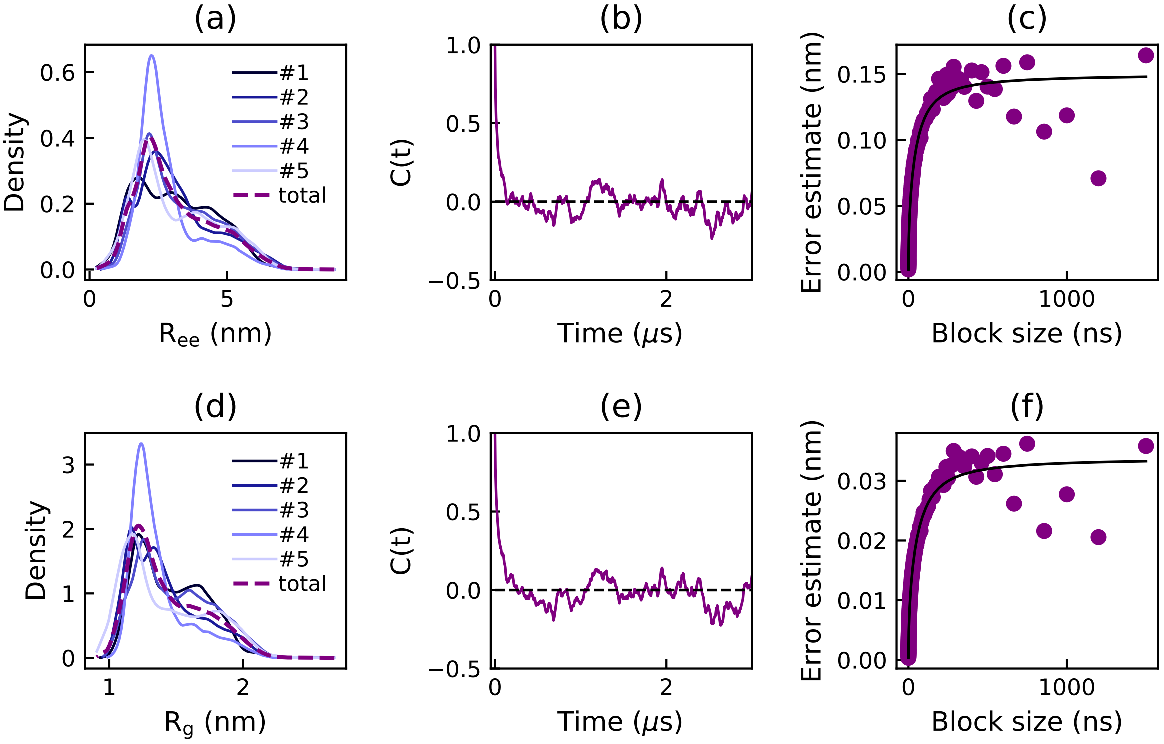

Figure S13: Density estimates of the end-to-end distance (R_{ee}) (a) and the radius of gyration (R_g) (d) for the five replicates and the concatenated simulation of bCPP in Amber ff99SB-ILDN, obtained from a Gaussian kernel estimator. Autocorrelation function (C(t)) and error estimate from block averaging of the end-to-end distance (b,c) and the radius of gyration (e,f) for the concatenated simulation.

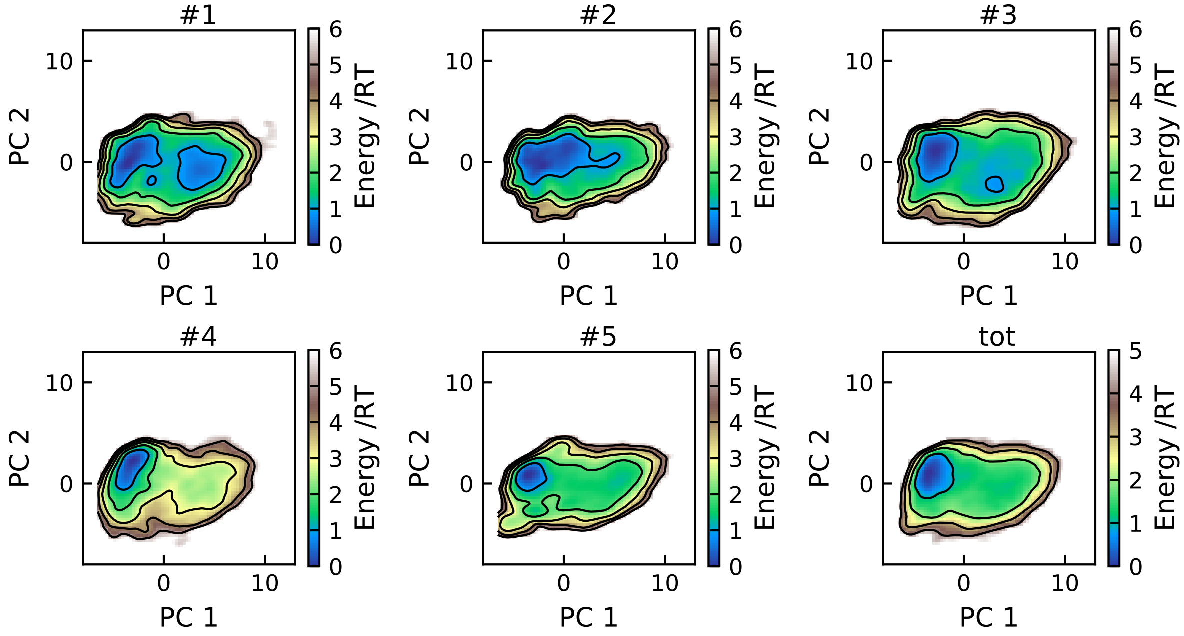

Figure S14: Energy landscapes for the five replicates and the concatenated trajectory of bCPP in Amber ff99SB-ILDN, using the first two principal components. All plots have been constructed using the same basis set and are therefore directly comparable. Contour lines are drawn for integer energy levels in the interval $1 \leq RT \leq 5$.

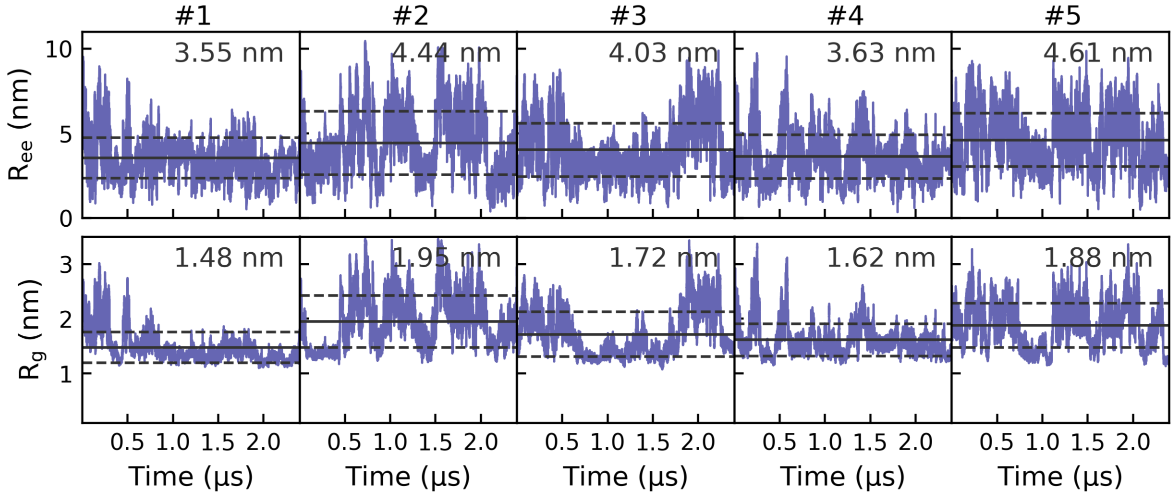

Figure S15: Time evolution of the end-to-end distance (R_{ee}) and the radius of gyration (R_g) for the five replicates in the simulation of Stath in Amber ff99SB-ILDN. The horizontal solid line represents the average in each replicate, with the dashed lines showing the standard deviation.

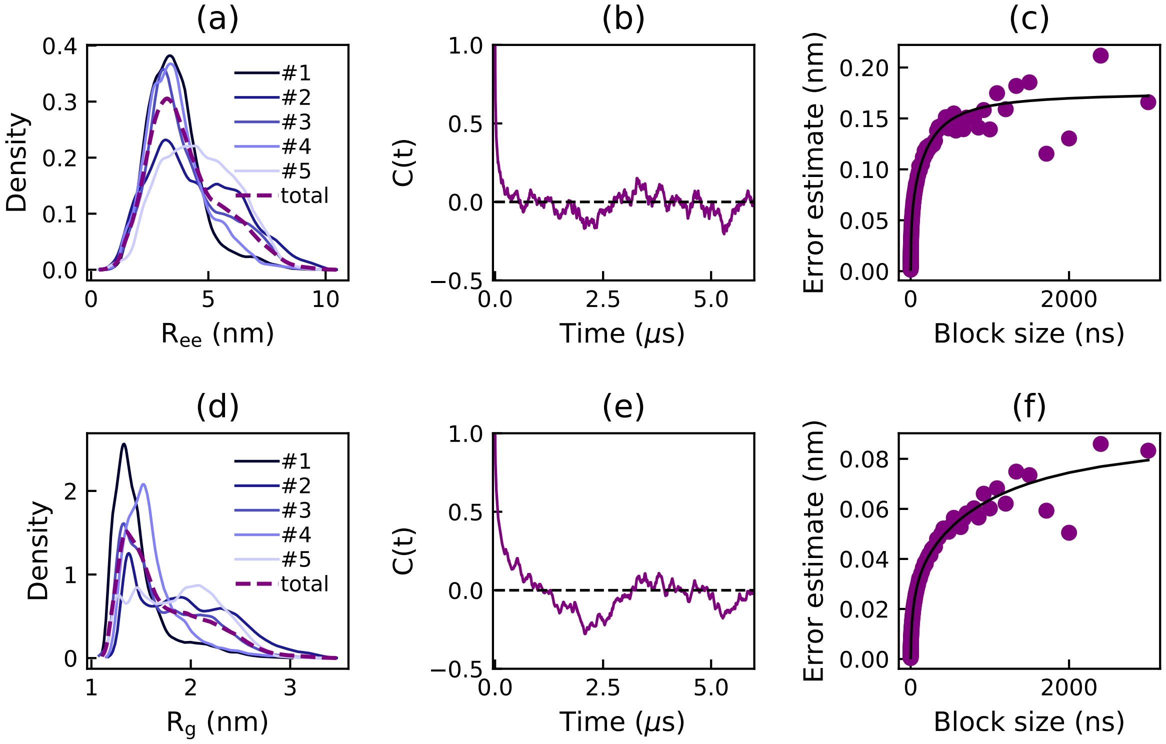

Figure S16: Density estimates of the end-to-end distance (R_{ee}) (a) and the radius of gyration (R_g) (d) for the five replicates and the concatenated simulation of Stath in Amber ff99SB-ILDN, obtained from a Gaussian kernel estimator. Autocorrelation function (C(t)) and error estimate from block averaging of the end-to-end distance (b,c) and the radius of gyration (e,f) for the concatenated simulation.

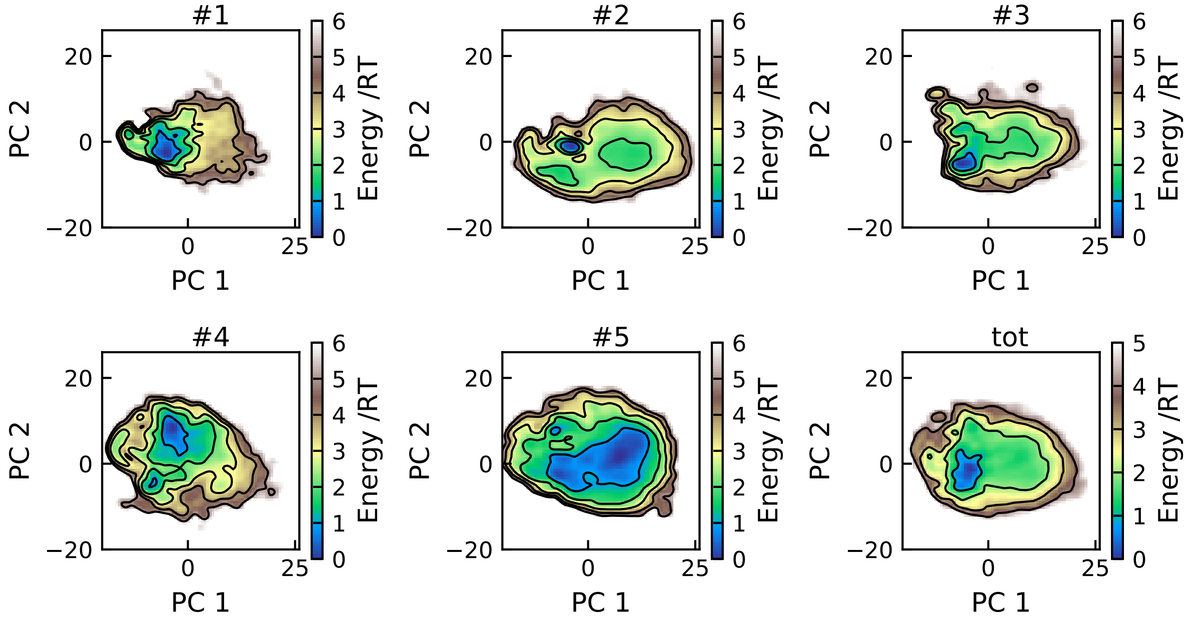

Figure S17: Energy landscapes for the five replicates and the concatenated trajectory of Stath in Amber ff99SB-ILDN, using the first two principal components. All plots have been constructed using the same basis set and are therefore directly comparable. Contour lines are drawn for integer energy levels in the interval $1 \leq RT \leq 5$.

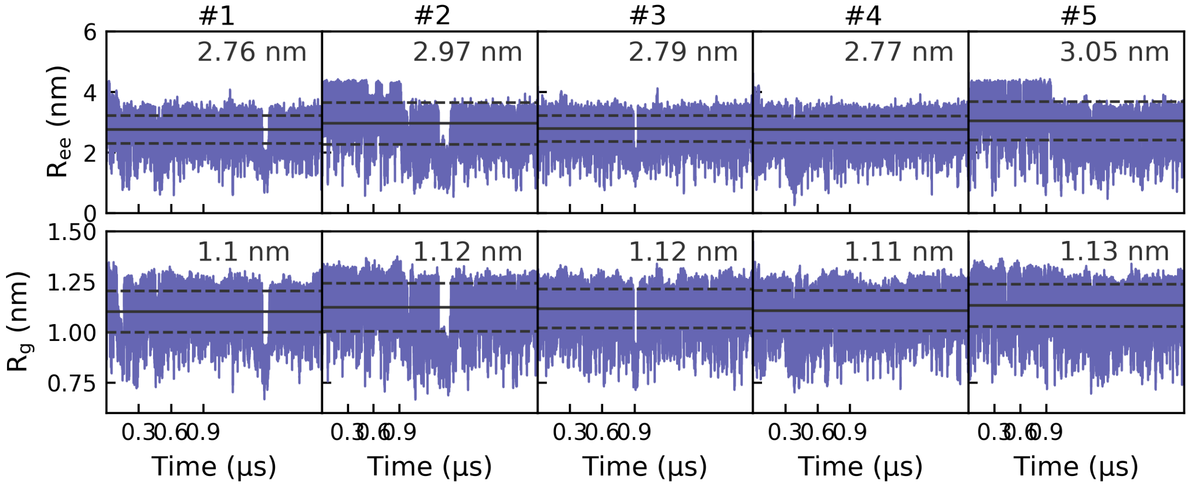

Figure S18: Time evolution of the end-to-end distance (R_{ee}) and the radius of gyration (R_g) for the five replicates in the simulation of Tau1 in CHARMM36m. The horizontal solid line represents the average in each replicate, with the dashed lines showing the standard deviation.

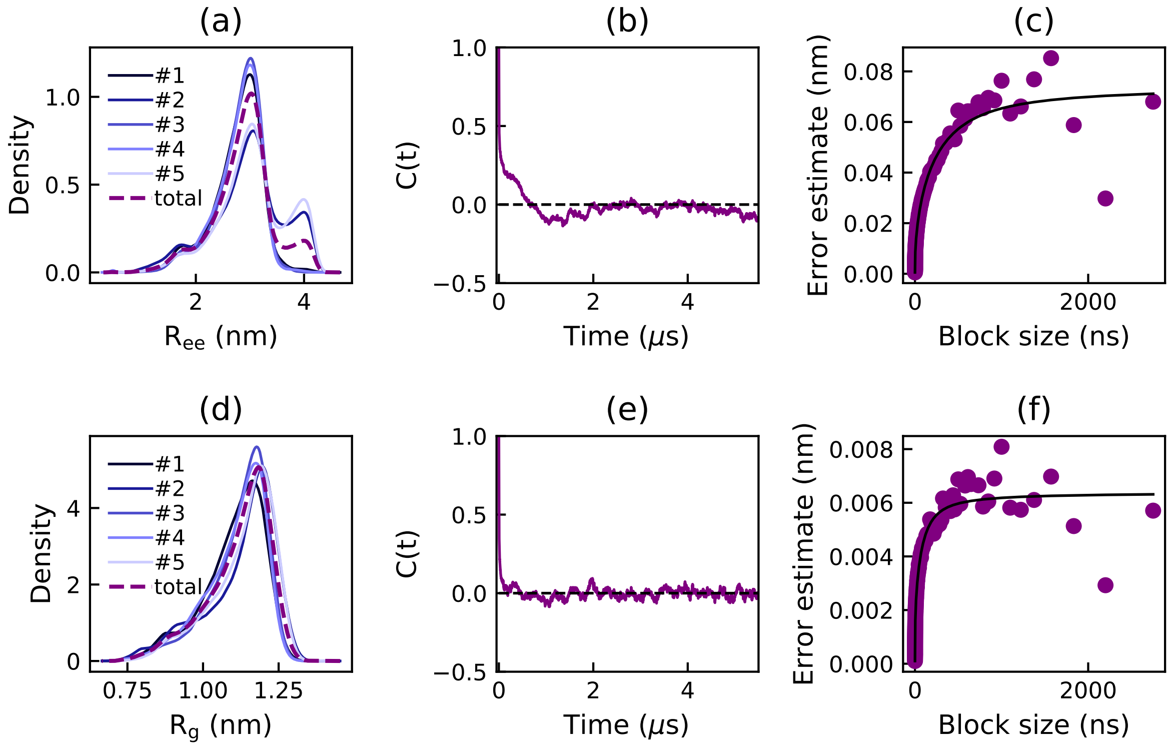

Figure S19: Density estimates of the end-to-end distance (R_{ee}) (a) and the radius of gyration (R_g) (d) for the five replicates and the concatenated simulation of Tau1 in CHARMM36m, obtained from a Gaussian kernel estimator. Autocorrelation function (C(t)) and error estimate from block averaging of the end-to-end distance (b,c) and the radius of gyration (e,f) for the concatenated simulation.

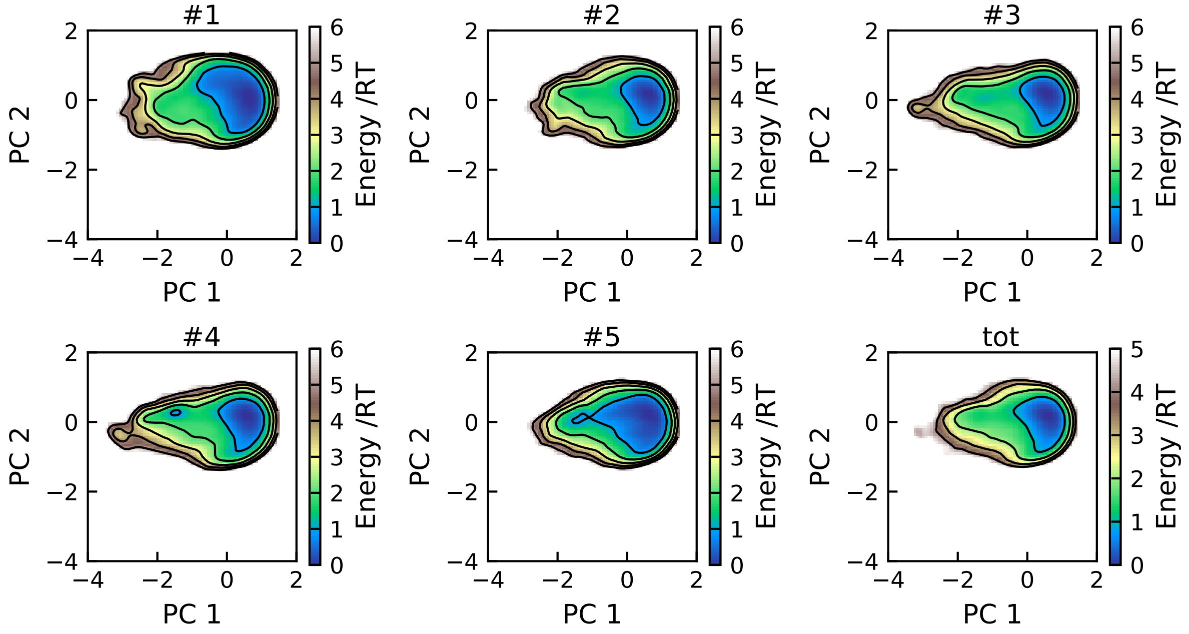

Figure S20: Energy landscapes for the five replicates and the concatenated trajectory of Tau1 in CHARMM36m, using the first two principal components. All plots have been constructed using the same basis set and are therefore directly comparable. Contour lines are drawn for integer energy levels in the interval $1 \leq RT \leq 5$.

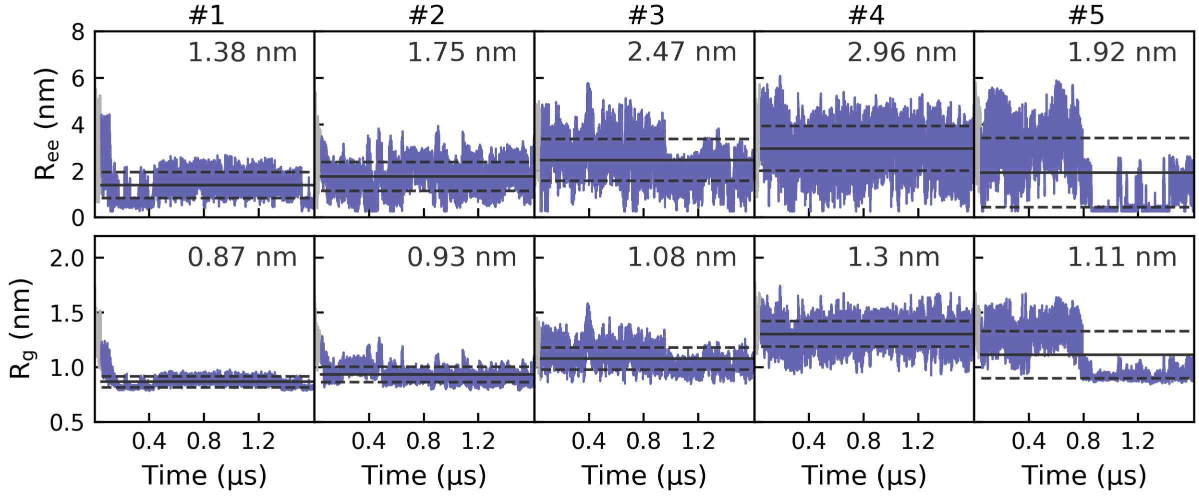

Figure S21: Time evolution of the end-to-end distance (R_{ee}) and the radius of gyration (R_g) for the five replicates in the simulation of Tau2 in CHARMM36m. The horizontal solid line represents the average in each replicate, with the dashed lines showing the standard deviation. The region removed before final analysis is plotted in gray.

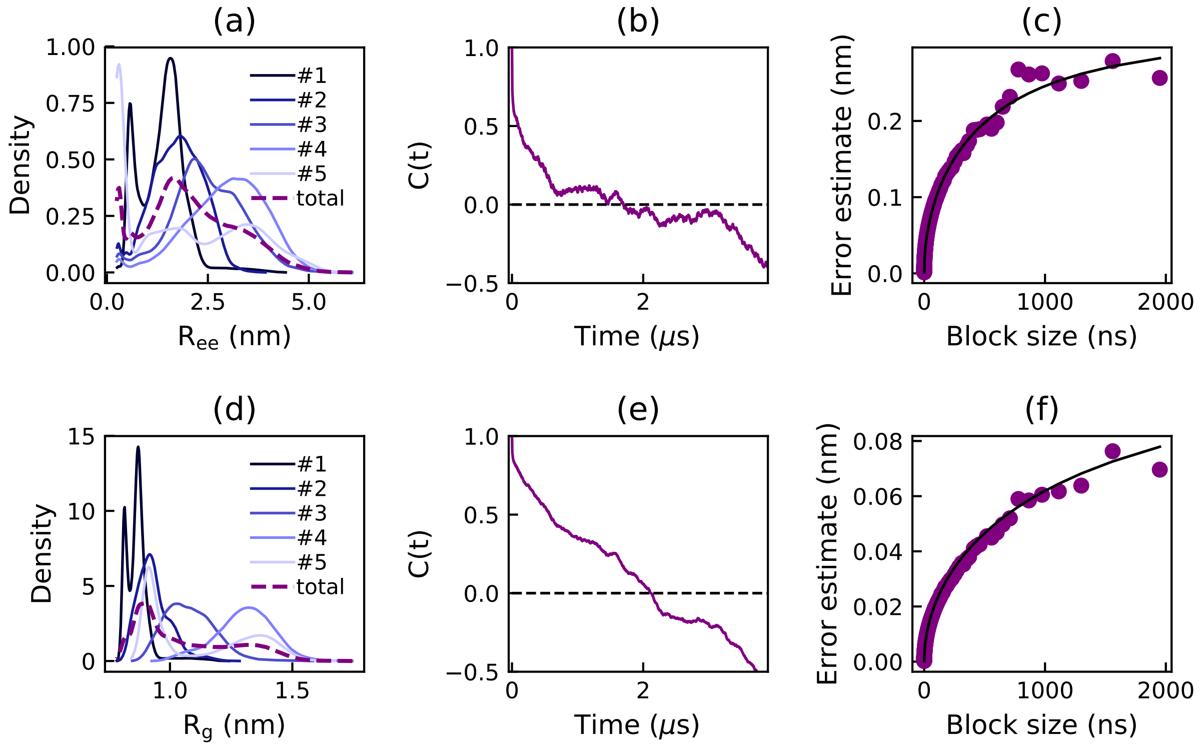

Figure S22: Density estimates of the end-to-end distance (R_{ee}) (a) and the radius of gyration (R_g) (d) for the five replicates and the concatenated simulation of Tau2 in CHARMM36m, obtained from a Gaussian kernel estimator. Autocorrelation function (C(t)) and error estimate from block averaging of the end-to-end distance (b,c) and the radius of gyration (e,f) for the concatenated simulation.

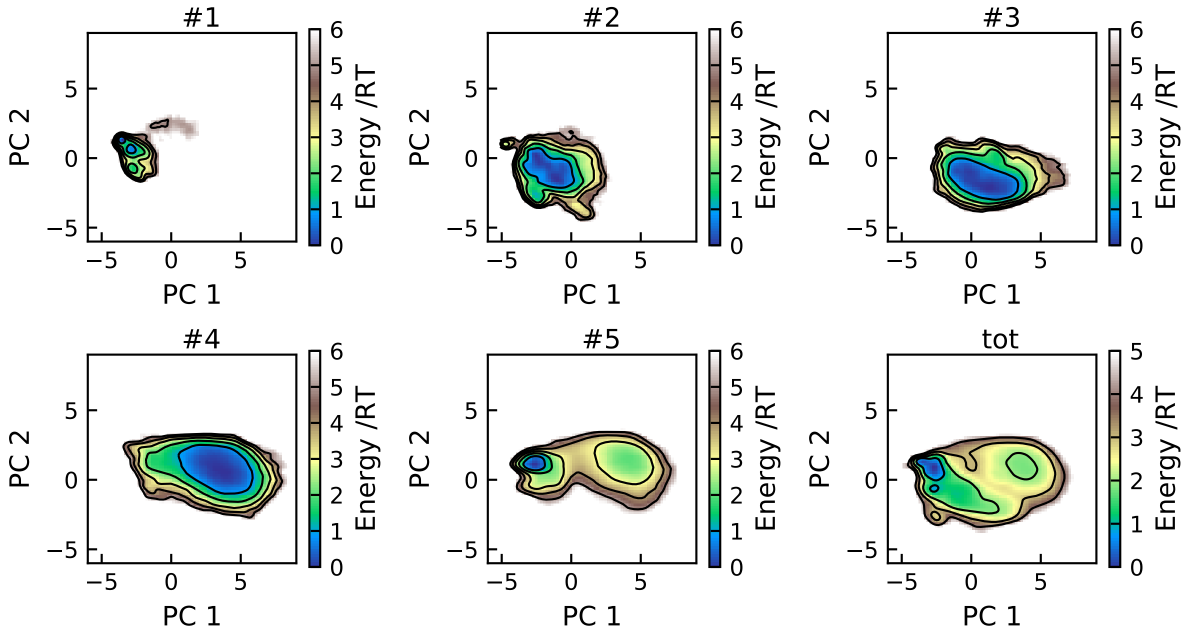

Figure S23: Energy landscapes for the five replicates and the concatenated trajectory of Tau2 in CHARMM36m, using the first two principal components. All plots have been constructed using the same basis set and are therefore directly comparable. Contour lines are drawn for integer energy levels in the interval $1 \leq RT \leq 5$.

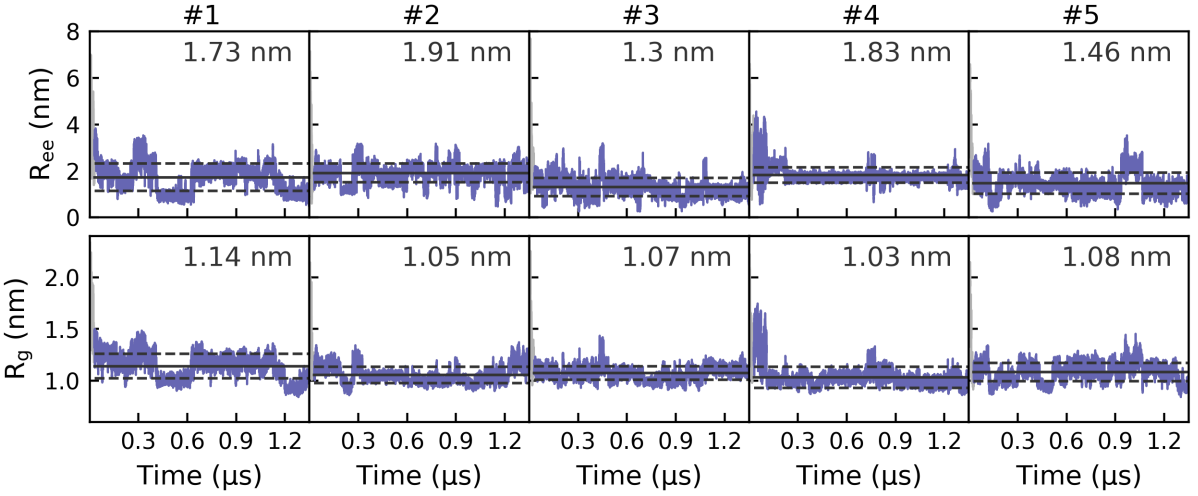

Figure S24: Time evolution of the end-to-end distance (R_{ee}) and the radius of gyration (R_g) for the five replicates in the simulation of bCPP in CHARMM36m. The horizontal solid line represents the average in each replicate, with the dashed lines showing the standard deviation. The region removed before final analysis is plotted in gray.

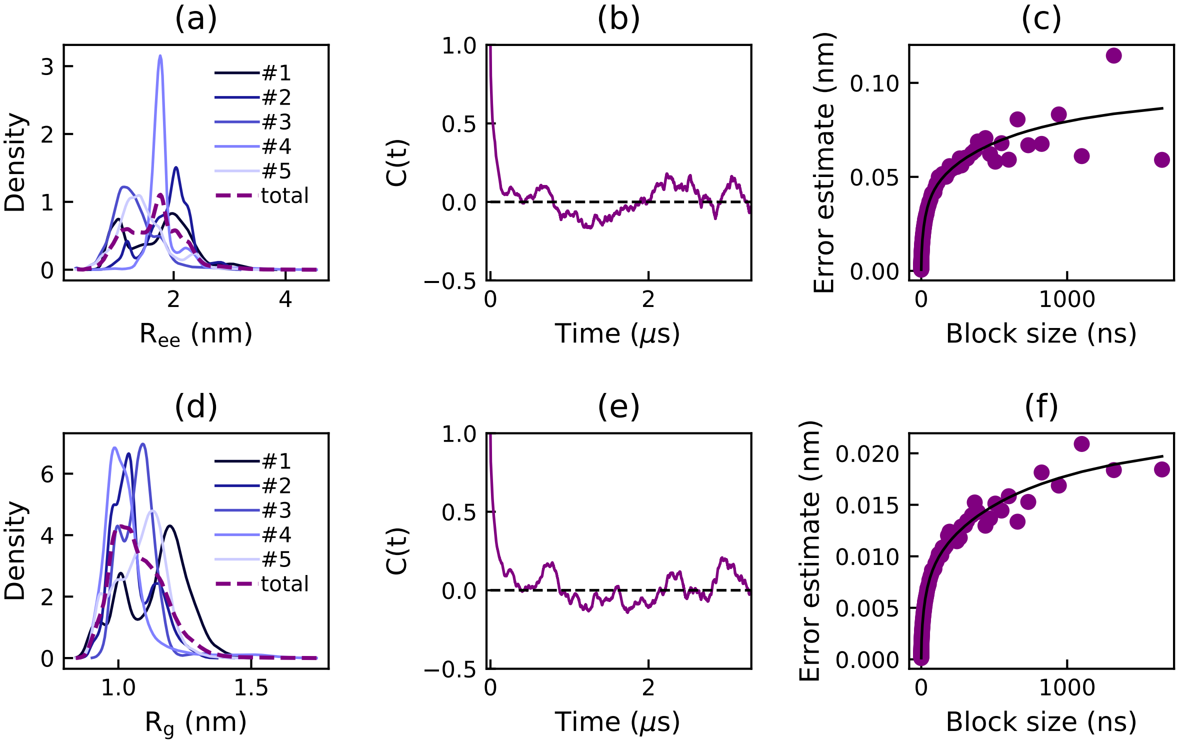

Figure S25: Density estimates of the end-to-end distance (R_{ee}) (a) and the radius of gyration (R_g) (d) for the five replicates and the concatenated simulation of bCPP in CHARMM36m, obtained from a Gaussian kernel estimator. Autocorrelation function (C(t)) and error estimate from block averaging of the end-to-end distance (b,c) and the radius of gyration (e,f) for the concatenated simulation.

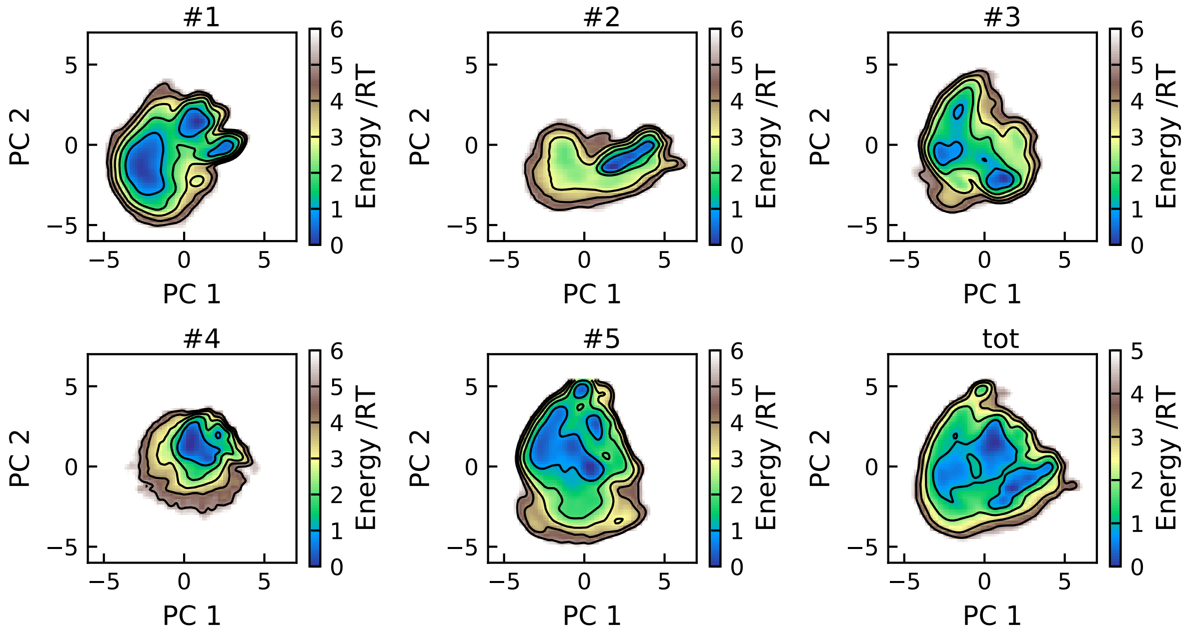

Figure S26: Energy landscapes for the five replicates and the concatenated trajectory of bCPP in CHARMM36m, using the first two principal components. All plots have been constructed using the same basis set and are therefore directly comparable. Contour lines are drawn for integer energy levels in the interval $1 \leq RT \leq 5$.

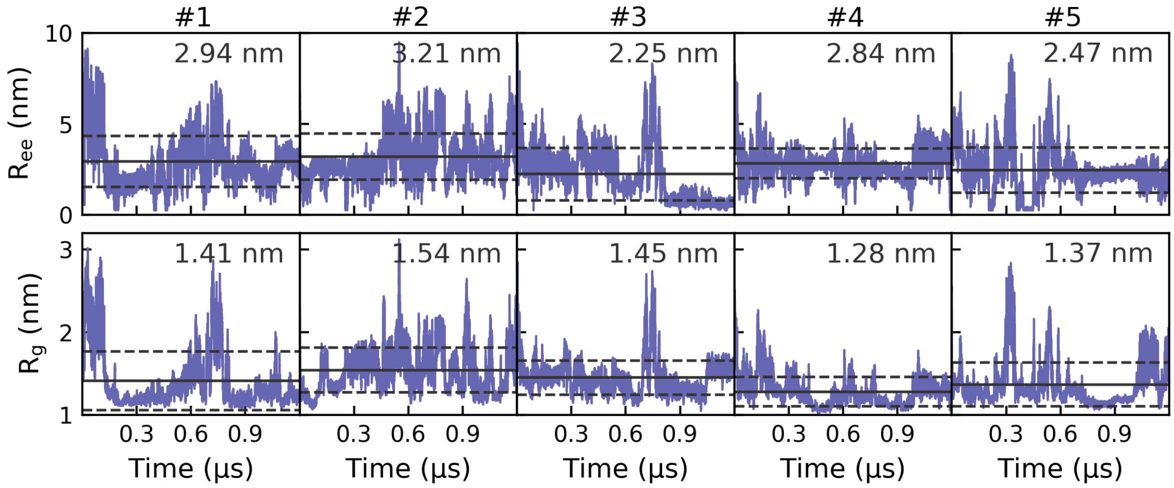

Figure S27: Time evolution of the end-to-end distance (R_{ee}) and the radius of gyration (R_g) for the five replicates in the simulation of Stath in CHARMM36m. The horizontal solid line represents the average in each replicate, with the dashed lines showing the standard deviation.

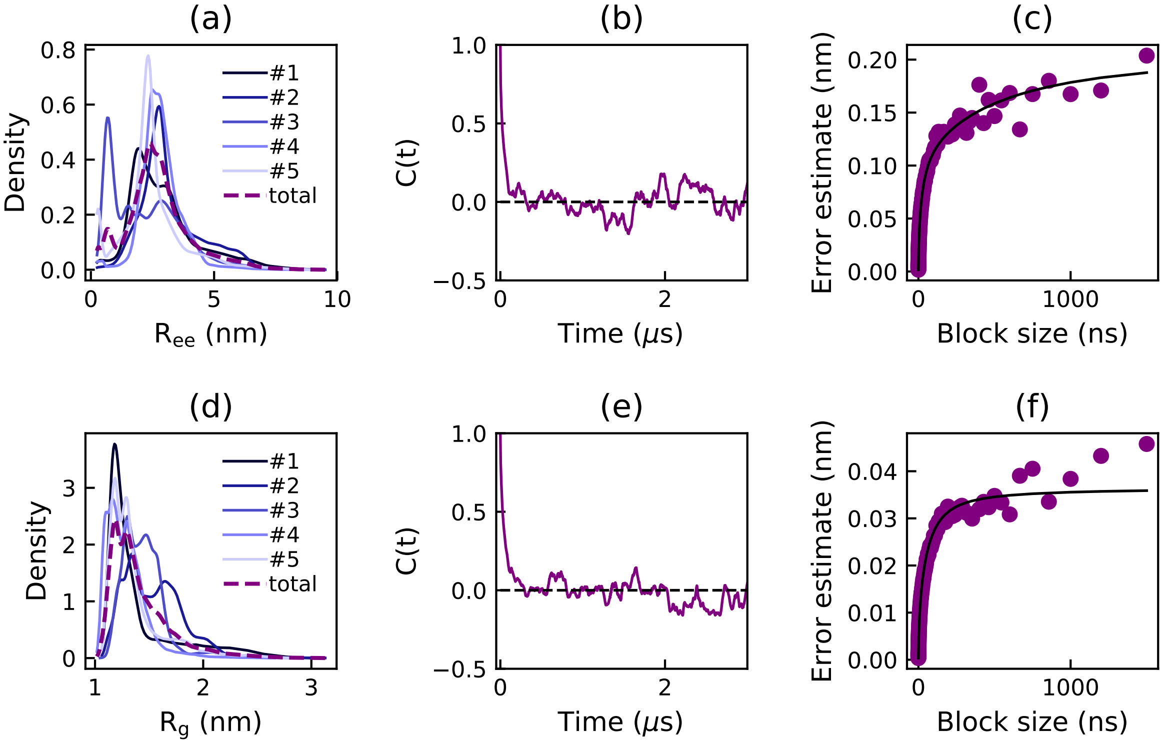

Figure S28: Density estimates of the end-to-end distance (R_{ee}) (a) and the radius of gyration (R_g) (d) for the five replicates and the concatenated simulation of Stath in CHARMM36m, obtained from a Gaussian kernel estimator. Autocorrelation function (C(t)) and error estimate from block averaging of the end-to-end distance (b,c) and the radius of gyration (e,f) for the concatenated simulation.

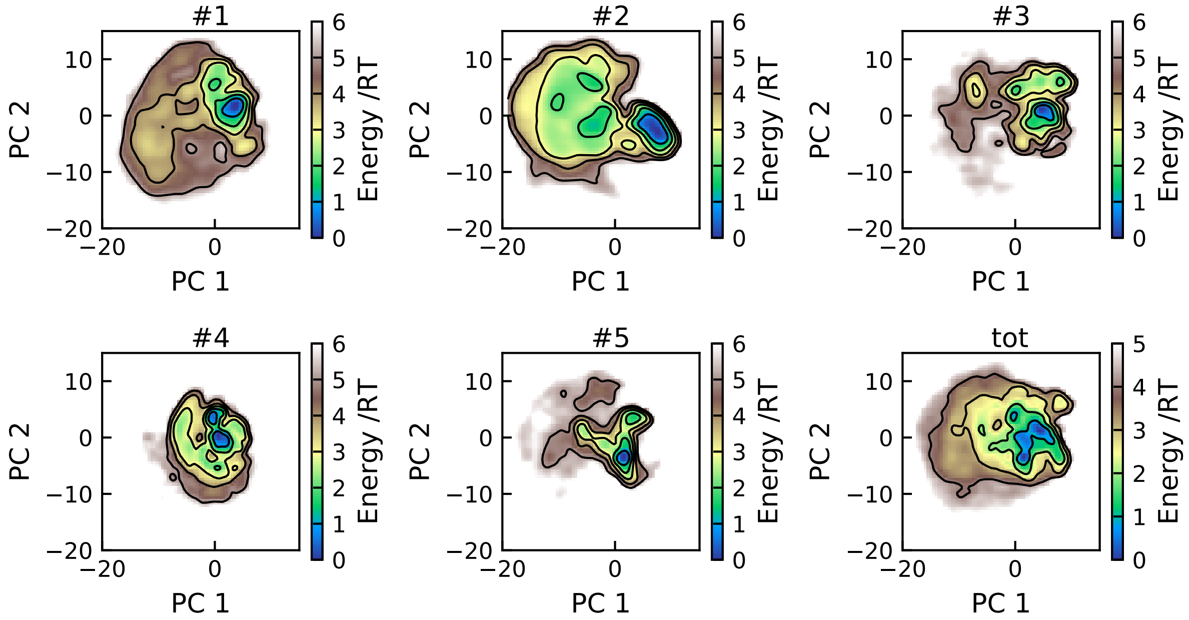

Figure S29: Energy landscapes for the five replicates and the concatenated trajectory of Stath in CHARMM36m, using the first two principal components. All plots have been constructed using the same basis set and are therefore directly comparable. Contour lines are drawn for integer energy levels in the interval $1 \leq RT \leq 5$.

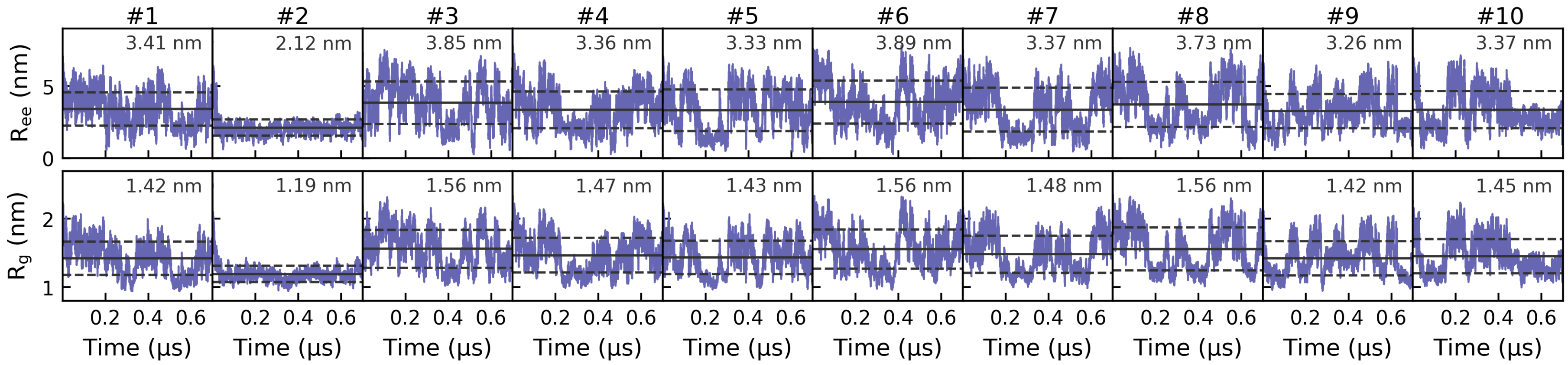

Figure S30: Time evolution of the end-to-end distance (R_{ee}) and the radius of gyration (R_g) for the ten replicates in the simulation of bCPP with 150 mM NaCl in Amber ff99SB-ILDN. The horizontal solid line represents the average in each replicate, with the dashed lines showing the standard deviation.

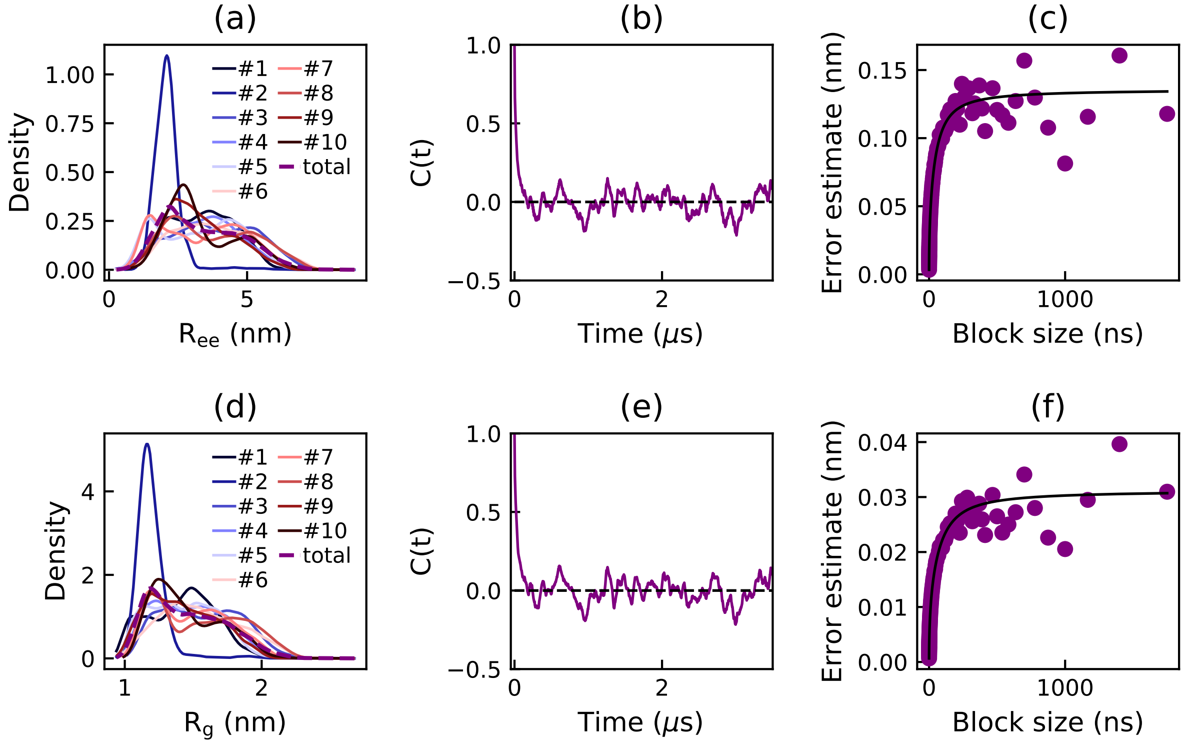

Figure S31: Density estimates of the end-to-end distance (R_{ee}) (a) and the radius of gyration (R_g) (d) for the ten replicates and the concatenated simulation of bCPP with 150 mM NaCl in Amber ff99SB-ILDN, obtained from a Gaussian kernel estimator. Autocorrelation function (C(t)) and error estimate from block averaging of the end-to-end distance (b,c) and the radius of gyration (e,f) for the concatenated simulation.

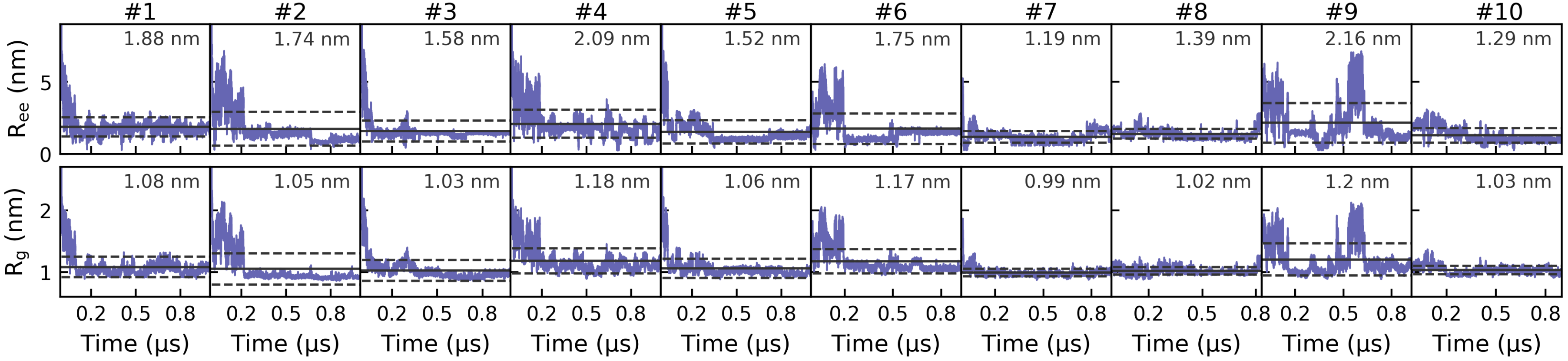

Figure S32: Time evolution of the end-to-end distance (R_{ee}) and the radius of gyration (R_g) for the ten replicates in the simulation of bCPP with 150 mM NaCl in CHARMM36m. The horizontal solid line represents the average in each replicate, with the dashed lines showing the standard deviation.

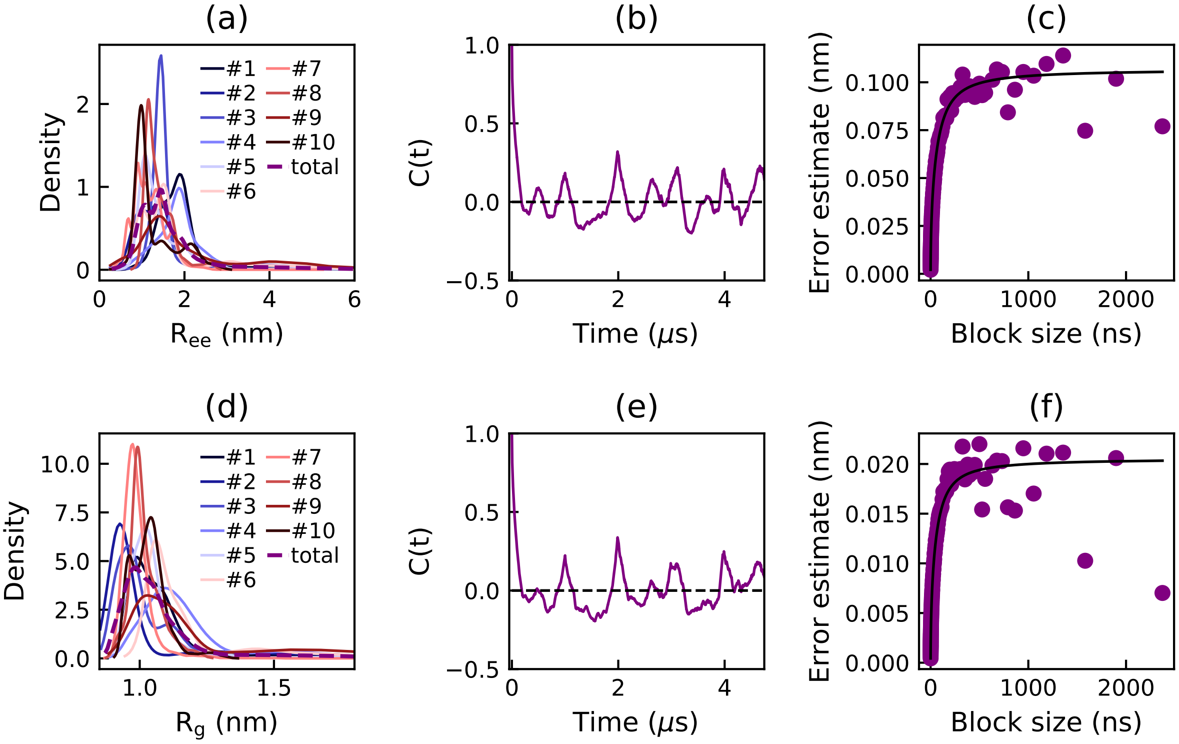

Figure S33: Density estimates of the end-to-end distance (R_{ee}) (a) and the radius of gyration (R_g) (d) for the ten replicates and the concatenated simulation of bCPP with 150 mM NaCl in CHARMM36m, obtained from a Gaussian kernel estimator. Autocorrelation function (C(t)) and error estimate from block averaging of the end-to-end distance (b,c) and the radius of gyration (e,f) for the concatenated simulation.

References

[1] D. Franke, M. V. Petoukhov, P. V. Konarev, A. Panjkovich, A. Tuukkanen, H. D. T. Mertens, A. G. Kikhney, N. R. Hajizadeh, J. M. Franklin, C. M. Jeffries, and D. I. Svergun. *ATSAS 2.8*: a comprehensive data analysis suite for small-angle scattering from macromolecular solutions. *J. Appl. Crystallogr.*, 50(4):1212–1225, Aug 2017.

[2] Carolina Cragnell, Ellen Rieloff, and Marie Skepö. Utilizing coarse-grained modeling and monte carlo simulations to evaluate the conformational ensemble of intrinsically disordered proteins and regions. *Journal of Molecular Biology*, 430(16):2478–2492, 2018.
